# Follicular Fluid-Derived Extracellular Vesicles Influence on In Vitro Maturation of Equine Oocyte: Impact on Cumulus Cell Viability, Expansion and Transcriptome

**DOI:** 10.3390/ijms25063262

**Published:** 2024-03-13

**Authors:** Julia Gabryś, Artur Gurgul, Tomasz Szmatoła, Barbara Kij-Mitka, Aneta Andronowska, Elżbieta Karnas, Mirosław Kucharski, Joanna Wojciechowska-Puchałka, Joanna Kochan, Monika Bugno-Poniewierska

**Affiliations:** 1 Department of Animal Reproduction, Anatomy and Genomics, Faculty of Animal Science, University of Agriculture in Krakow, Mickiewicza 24/28, 30-059 Krakow, Poland; julia.gabrys@urk.edu.pl (J.G.); barbara.kij-mitka@urk.edu.pl (B.K.-M.); joanna.wojciechowska-puchalka@urk.edu.pl (J.W.-P.); joanna.kochan@urk.edu.pl (J.K.); monika.bugno-poniewierska@urk.edu.pl (M.B.-P.); 2 Center for Experimental and Innovative Medicine, University of Agriculture in Krakow, Rędzina 1c, 30-248 Krakow, Poland; tomasz.szmatola@urk.edu.pl; 3 Institute of Animal Reproduction and Food Research, Polish Academy of Sciences, Tuwima 10, 10-748 Olsztyn, Poland; a.andronowska@pan.olsztyn.pl; 4 Department of Cell Biology, Faculty of Biochemistry, Biophysics and Biotechnology, Jagiellonian University, Gronostajowa 7, 30-387 Krakow, Poland; e.karnas@uj.edu.pl; 5 Department of Animal Physiology and Endocrinology, University of Agriculture in Krakow, Mickiewicza 24/28, 30-059 Krakow, Poland; miroslaw.kucharski@urk.edu.pl

**Keywords:** extracellular vesicles, follicular fluid, mare, cumulus cells, in vitro maturation, assisted reproductive techniques, transcriptome, expansion, viability, cumulus–oocyte complex

## Abstract

Cumulus cell (CC) expansion is pivotal for oocyte maturation, during which CCs release factors that initiate paracrine signaling within the follicular fluid (FF). The FF is abundant in extracellular vesicles (EVs) that facilitate intercellular communication. Although bovine and murine EVs can control cumulus expansion, these effects have not been observed in equines. This study aimed to assess the impact of FF-derived EVs (ffEVs) on equine CC expansion, viability, and transcriptome. Cumulus–oocyte complexes (COCs) that underwent in vitro maturation (IVM) in the presence (200 µg protein/mL) or absence (control) of ffEVs were assessed for cumulus expansion and viability. CCs were isolated after 12 h of IVM, followed by RNA extraction, cDNA library generation, and subsequent transcriptome analysis using next-generation sequencing. Confocal microscopy images illustrated the internalization of labeled ffEVs by CCs. Supplementation with ffEVs significantly enhanced cumulus expansion in both compacted (Cp, *p* < 0.0001) and expanded (Ex, *p* < 0.05) COCs, while viability increased in Cp groups (*p* < 0.01), but decreased in Ex groups (*p* < 0.05), compared to the controls. Although transcriptome analysis revealed a subtle effect on CC RNA profiles, differentially expressed genes encompassed processes (e.g., MAPK and Wnt signaling) potentially crucial for cumulus properties and, consequently, oocyte maturation.

## 1. Introduction

The process of intercellular communication within the ovarian follicle microenvironment is the basic mechanism that enables oocytes to develop [1]. Oocyte development involves cytoplasmic, nuclear, and molecular maturation, which require communication between the oocyte and surrounding cumulus cells (CC), as well as cumulus expansion [2,3].

Cumulus cells (CCs) are a crucial part of the cumulus–oocyte complex (COC), which envelopes the oocyte. These cells affect the acquisition of competencies by the oocyte, including its ability to be fertilized, and its development to the blastocyst stage [4]. Following ovulation, intercellular connections between the CCs and the oocyte are broken, but the process of cumulus expansion enables continued association of the CCs to the oocyte throughout the ovulatory processes and during subsequent fertilization [5]. Cumulus expansion is characterized by the secretion of the hyaluronic acid-rich extracellular matrix by the CCs, and the expression of other proteins necessary for the formation and retention of the matrix [6,7]. Numerous factors control this process, including epidermal growth factor (EGF) as well as the expression of several genes, including prostaglandin endoperoxide synthase 2 (*PTGS2*), hyaluronan synthase 2 (*HAS2*), and pentraxin-related protein 3 (*PTX3*) [8]. Cumulus expansion promotes oocyte maturation and follicular wall puncture during ovulation, while a lack of expansion results in a lower rate of ovulation and subsequent female infertility [9,10]. Therefore, the number of CC layers and the extent of their expansion, as well as apoptosis, are extremely important parameters for assessing COC quality.

Follicular fluid (FF), which is secreted between the granulosa cell layers in antral follicles [11], plays a crucial role in ovarian steroidogenesis and oogenesis [12]. The major components of FF are electrolytes, steroid hormones, metabolites, proteins, polysaccharides, reactive oxygen species (ROS), antioxidant enzymes, and various molecules including non-coding RNAs, which provide the path of autocrine and paracrine communication between the theca, mural, and cumulus cells and the maturing oocyte [13]. Follicular fluid has a variety of oocyte-related functions such as maintenance of meiotic arrest [14], protection against proteolysis [15], as well as aiding in the development and maturation of oocyte and cumulus cells [16]. Recent studies have shown that FF also contains extracellular vesicles (EVs), which can be involved in intercellular communication in the microenvironment of the ovarian follicle [17,18,19].

EVs are a heterogeneous group of membrane-bound nanoparticles secreted by various types of cells, which are capable of influencing cell response and bioactivity by delivering molecular signals and transmitting proteins, messenger RNAs (mRNAs), and non-coding RNAs as microRNAs (miRNAs) between donor and recipient cells [20]. The components of EVs are transferred to the recipient cells via internalization and can serve as a biomarker and an indication of the involvement of EVs in biological processes [21,22,23,24]. Formerly categorized as exosomes/microvesicles, EVs present challenges in terms of their purification and identification. Therefore, MISEV guidelines (Minimal Information for Studies of Extracellular Vesicles) established a framework enabling EV characterization based on size (<200 nm—“small EVs”; >200 nm—“medium/large EVs”), cell of origin, biochemical composition, or density [25].

One of the bioactive components of EVs are microRNAs (miRNAs). These short non-coding sequences can regulate gene expression by directly targeting the mRNA and inhibiting its translation to a protein, or/and by interfering with the epigenome [26,27]. Therefore, miRNAs are capable of modulating biological pathways involved in folliculogenesis [28], and extracellular miRNAs (exmiRNAs) from FF are predicted to influence targets that regulate the epidermal growth factor receptor (EGFR), transforming growth factor beta (TGFβ), mitogen-activated protein kinase (MAPK), and Wnt signaling pathways, all of which are known to contribute to follicular development, oocyte meiotic resumption, and ovulation [29,30,31,32]. Since the majority of FF miRNAs are present within EVs [32], it is believed that follicular fluid EVs (ffEVs) play an important role in mediating cell-to-cell communication within the follicular microenvironment and may modulate biological signaling pathways via transferring bioactive molecules such as miRNAs.

The manner of interaction and physiological homogeneity of EVs are currently the subject of many studies in relation to folliculogenesis, gametogenesis, and embryo development in diverse animal species [29,33,34,35]. Several studies in mice, pigs, and cattle have shown that EVs can significantly affect changes in the transcriptome during gamete and embryo development [35,36,37]. Moreover, in recent years, EVs extracted from FF have been shown to affect the gene expression [32,38], expansion [39], and proliferation [40] of cultured bovine CCs.

While there have been reports on the composition of miRNAs and proteins within ffEVs, along with their internalization by CCs of the domestic horse [19,30], there is still no evidence of the cellular and transcriptomic modulations of equine CCs following supplementation of the in vitro maturation (IVM) medium with ffEVs. Based on these results, our hypothesis is that ffEVs may affect the transcriptomic profile and physiology of CCs surrounding the oocyte during IVM. The aim of this study was to determine the effect of supplementation of IVM medium with ffEVs upon equine CC viability, expansion, and transcriptome. The gene expression differences (DEGs) between supplemented and control groups were investigated.

## 2. Results

### 2.1. ffEV Characterization

**Figure 1:**
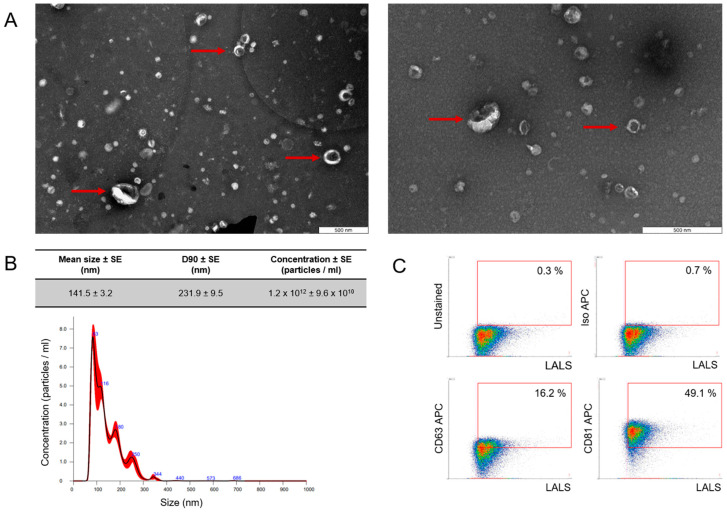
Characterization of follicular fluid-derived EVs. (**A**) EVs purified from follicular fluid were analyzed by transmission electron microscopy, where the red arrow points toward the presence of EVs. (**B**) The size profile was measured by NanoSight NTA, which revealed one population of ffEVs with a mean diameter of 141.5 nm. D90—value indicates the percentage of particles (90%) less than or equal to the appropriate mean particle size. The red bars represent the standard error of the mean, while the blue numbers denote the means of clusters. (**C**) Density plot of EVs purified from follicular fluid showed a positive signal for EV-specific markers CD63 and CD81. To validate the specificity of the data obtained, APC isotype and unstained control were incorporated into the gating strategy.

FF-derived EVs were characterized using nanoparticle tracking analysis (NTA), transmission electron microscopy (TEM), and flow cytometry (FC). Physical analysis using TEM showed the presence of particles with the cup shape that is characteristic of EVs, as indicated by the arrows (Figure 1A). NTA showed that most of the molecules obtained were within the limits of “small EVs” [25], with a mean particle size of 141.5 nm, and a D90 value of 231.9 nm, signifying that 90% of the particles were equal to or smaller than the appropriate mean particle size [25] (Figure 1B). The mean EV concentration was 1.2 × 10^12^ +/− 9.6 × 10^10^ and the efficacy in acquiring EVs from follicular fluid reached approximately 6 × 10^9^ particles/mL. Phenotypical analysis with high-resolution FC confirmed that the isolated particles expressed EV-specific surface markers (Figure 1C) at the level of 16.2% and 49.1% for CD63 and CD81, respectively.

### 2.2. ffEV Labeling and Uptake by COCs

**Figure 2:**
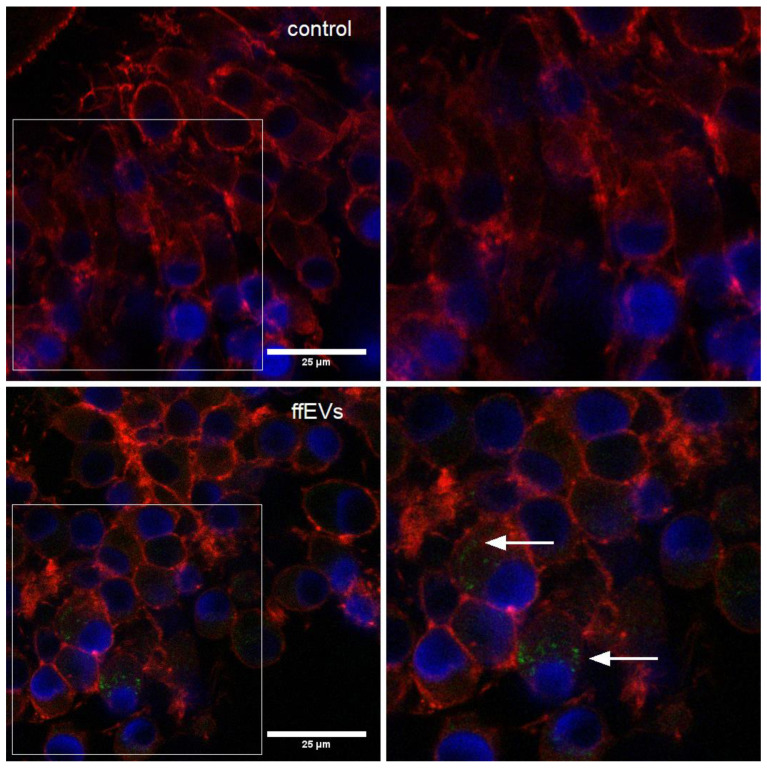
The uptake of labeled ffEVs by cumulus cells (CCs) in COC in in vitro culture was visualized using a Leica SP8 WRL scanning confocal microscope. Internalized ffEVs manifest as green spots within the actin filaments (red) in the inner layers of CCs. Cell nuclei, stained with Hoechst 33342, appear in blue. The arrows indicate the presence of ffEVs inside the CCs.

The evaluation of ffEV uptake by COCs was conducted following a 38 h maturation period. Upon fixation and microscopic analysis, the presence of labeled ffEVs in the cytoplasm of CCs surrounding maturing oocytes was noted in both compacted (Cp) and expanded (Ex) COCs. No internalization was observed in the negative control (Figure 2).

### 2.3. Cumulus Expansion

**Figure 3:**
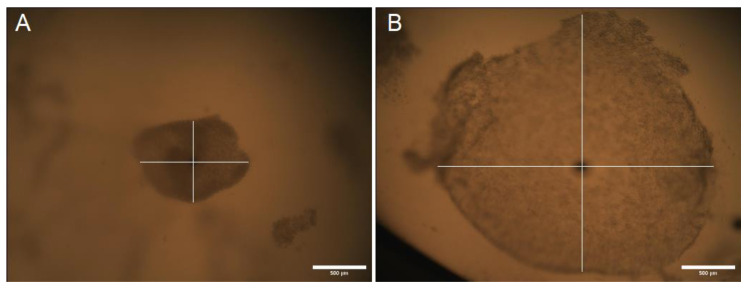
Equine ffEVs induce expansion of the cumulus oophorus. COCs were matured in vitro with ffEVs from small follicles. COCs were imaged by OptaView at the beginning of culture (0 h; (**A**)) and following in vitro maturation (38 h; (**B**)). The measurement of the COC dimensions is illustrated by the intersecting white lines.

**Figure 4:**
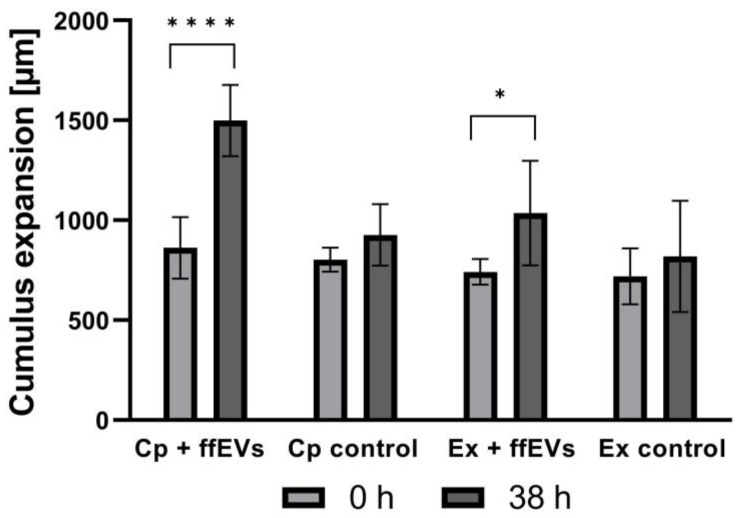
Equine ffEVs induce cumulus expansion of equine COCs. COCs were measured in ImageJ at the beginning of culture (0 h) and following in vitro maturation (38 h). Negative control COCs, both Cp and Ex, cultured without ffEVs did not expand significantly (*p* > 0.05). However, both Cp and Ex COCs cultured with small-follicle EVs exhibited increased cumulus expansion. Means with asterisks are significantly different (one asterisk, *p* < 0.05; four asterisks, *p* < 0.0001).

Cumulus expansion was determined by analysis of alterations in the dimensions (µm) of 204 COCs before and after IVM was conducted (Figure 3). At the end of the IVM period, both the Cp and Ex COCs supplemented with ffEVs showed a greater expansion of the cumulus oophorus compared to those from control groups. Average COC size (µm) at 0 h and 38 h confirmed that ffEVs from small follicles (<20 mm) induced significantly greater cumulus expansion than occurred in medium alone (Figure 4). What is more, ffEV supplementation of Cp COCs exhibited a greater effect on cumulus expansion than supplementation of Ex COCs (*p* = 0.00006 vs. *p* = 0.01569). There were no significant differences in the size before and after IVM of either Cp or Ex COCs from the control groups (*p* = 0.144 vs. *p* = 0.453, respectively). The average change in COC dimensions is shown in Table S1.

### 2.4. Viability

**Figure 5:**
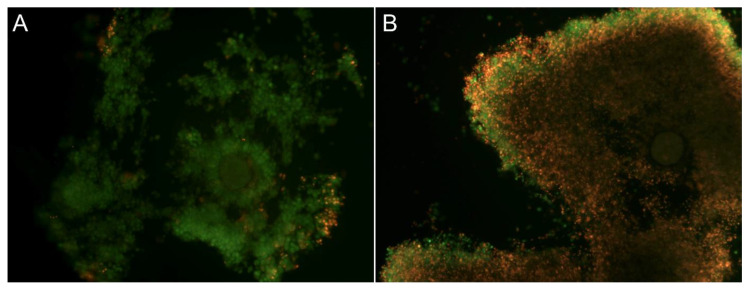
Equine ffEVs affect the viability of cumulus after maturation. COCs were imaged by OptaView 7.1 software on a Nikon Eclipse E600 fluorescence microscope after in vitro maturation (38 h). Representative example of COCs with high (**A**) and low (**B**) cell viability (97.5% and 57%, respectively). Green color denotes live, while red indicates dead cells. Magnification ×10.

**Figure 6:**
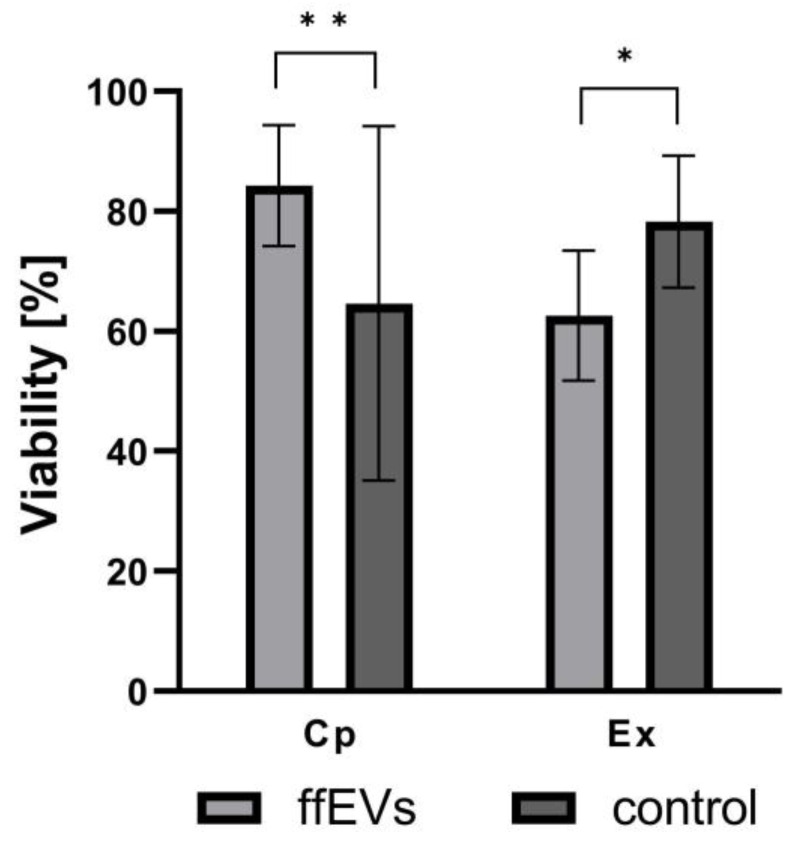
Equine ffEVs affect the viability of cumulus after maturation. COCs were imaged by OptaView 7.1 software on a Nikon Eclipse E600 fluorescence microscope after in vitro maturation (38 h). Cp COCs supplemented with ffEVs showed increased cumulus viability compared to the control group (*p* < 0.01). Negative-control Ex COCs exhibited increased cumulus viability compared with those cultured with ffEVs (*p* < 0.05). Means with asterisks are significantly different (one asterisk, *p* < 0.05; two asterisks, *p* < 0.01).

To evaluate the viability rate, 129 COCs were matured for 38 h in vitro in the presence (ffEVs) or absence (control) of ffEVs, and then stained (Figure 5). The addition of ffEVs had a significant impact on cumulus cell viability. While Cp COCs matured in vitro with the addition of ffEVs had a significantly higher average cumulus viability rate than the control group (84.3% vs. 64.7%, respectively; *p* = 0.002), Ex COCs supplemented with ffEVs had significantly lower cumulus viability than the control group (62.6% vs. 78.3% respectively; *p* = 0.013) (Figure 6).

### 2.5. Mapped Reads, Statistics and Global Expression Profiles

**Figure 7:**
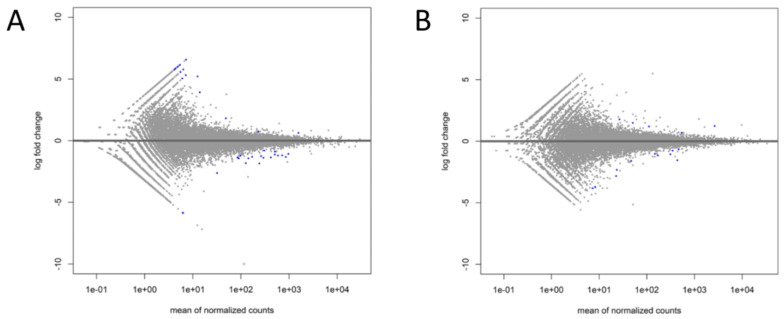
MA plots presenting log2fold change against the mean gene expression level of CCs from Cp (**A**) and Ex (**B**) COCs supplemented with ffEVs. Blue dots mark significantly DE genes (FDR < 0.1).

**Figure 8:**
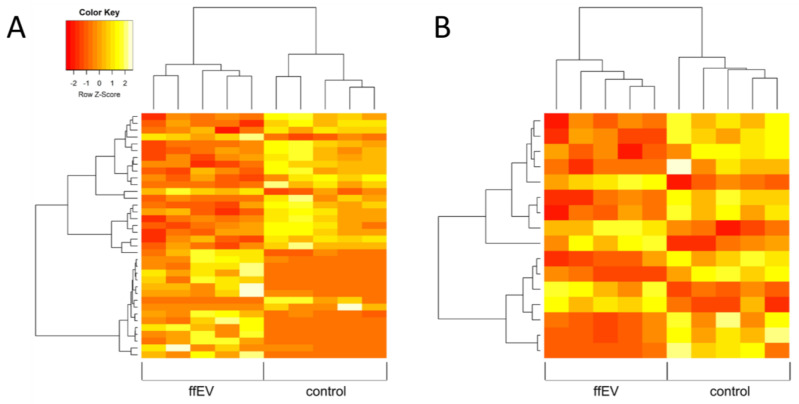
Expression profiles based on DE genes between the control group and ffEV-treated group in (**A**) Cp and (**B**) Ex COCs (FDR < 0.5).

**Figure 9:**
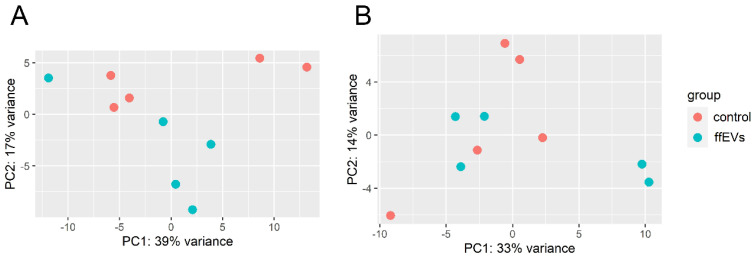
Principal component analysis of the DE gene profiles for CC in Cp (**A**) and Ex (**B**) COCs supplemented with ffEVs (blue) and control (red) (FDR < 0.05).

Approximately 6.41 × 10^6^ to 12.05 × 10^6^ reads after rimming were generated per sample during sequencing, with a median of 6.8 × 10^6^ reads. The mean mapping efficiency against the reference genome for all analyzed samples exceeded 63%, and 5% of reads were mapped to multiple loci (Table S2). The differences in the expression profiles between control and ffEV-supplemented groups revealed 36 transcripts that differed significantly for Cp COCs (16 upregulated and 20 downregulated genes) and 16 for Ex COCs (5 upregulated and 11 downregulated genes) (FDR < 0.1, Figure 7, Table S3). Genes with the most changed expression included DNA damage-inducible transcript 4 (*DDIT4*), dual specificity phosphatase 1 (*DUSP1*), high mobility group box 2 (*HMGB2*), and cyclin-dependent kinase inhibitor 1C (*CDKN1C*) (Table 1). With the use of hierarchical clustering with the differentially expressed (DE) genes, the expression profiles of the samples were categorized into distinct groups, implying a partially unique pattern of alterations in transcript expression among the analyzed groups (Figure 8). However, the overall evaluation of gene expression profiles (with all expressed genes) by principal component analysis led to an unclear separation of the groups (Figure 9).

### 2.6. Transcriptome Alterations in CC Supplemented with ffEVs during IVM

In Cp COCs, the genes differentially regulated between ffEV-supplemented and control CCs did not enrich after correction for multiple testing any of KEEG pathways, but showed some statistical trend toward the enrichment of the “endocytosis” (FDR = 0.067) (*FOS*, *KIF5C* genes) pathway, which is a crucial process in EV internalization, as well as the “MAPK signaling” (FDR = 0.079) (*FOS*, *DUSP1*) and “apoptosis” (FDR = 0.13) (*FOS*) pathways.

**Figure 10:**
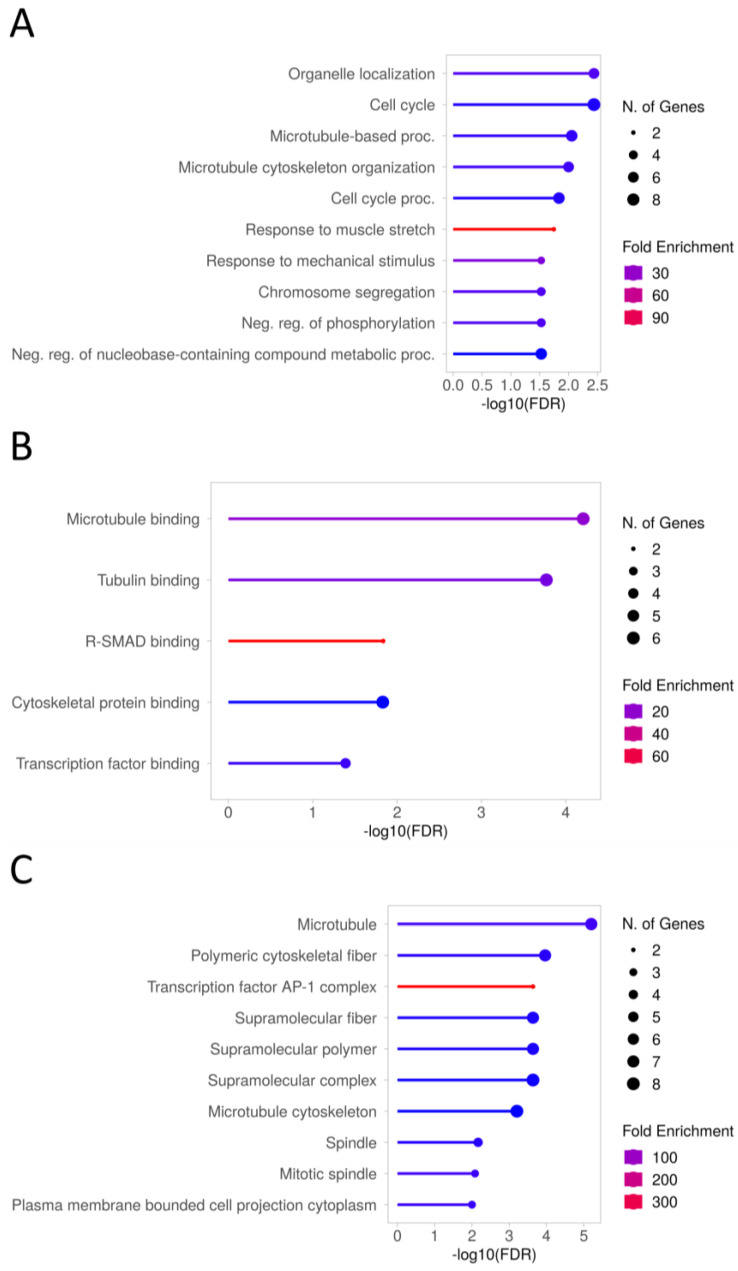
Selected biological processes (**A**), molecular functions (**B**), and cellular components (**C**) enriched in DE genes (adjP < 0.1) in CC of Cp COCs after 12 h treatment with ffEVs.

When examining the genes that showed differential expression between cells supplemented with ffEV and the control group, the analysis of associated gene ontology (GO) biological processes (using the *Equus caballus* reference panel in the ShinyGO 0.77 system) revealed a significant (FDR < 0.05) enrichment in categories related to the cell cycle (GO:0007049) (*GPNMB*, *PARD6B*, *NUSAP1*, *SEPTIN9*, *ASPM*, *JUN*, *CENPF*, *KIFC1*, *GADD45A)*, microtubule-based processes (GO:0007017) (*PARD6B*, *CEP57*, *KIF5C*, *NUSAP1*, *GAS8*, *ASPM*, *KIFC1)*, or chromosome segregation (GO:0007059) (*CIAO2A*, *NUSAP1*, *CENPF*, *KIFC1)*. Of the most enriched GO molecular functions, the most interesting seemed to be categories such as microtubule and tubulin binding (GO:0008017, GO:0015631) (*CEP57*, *KIF5C*, *NUSAP1*, *GAS8*, *CENPF*, *KIFC1*). In the case of enriched GO cellular components, the most important term appears to be microtubule (GO:0005874) (*CEP57*, *KIF5C*, *NUSAP1*, *GAS8*, *SEPTIN9*, *ASPM*, *KIFC1)* (Figure 10, Table S4). The analysis of DE genes using the PANTHER system revealed significant over-representation (*p*-value < 0.05) in similar GO-Slim categories, but only when results were not corrected for multiple testing. Within the most over-represented biological processes, the following were prominently featured: microtubule-based processes (GO:0007017) (*KIFC1*, *KIF5C*, *PARD6B*, *NUSAP1*), cytokinesis (GO:0000910) (*SEPTIN9*, *NUSAP1)*, or terms connected with regulation of the p38MAPK cascade (GO:0032873, GO:1900744) (*DUSP1*). Similarly, the over-represented molecular functions were represented by the terms connected with microtubule binding (GO:0008017) and microtubule motor activity (GO:0003777) (*KIFC1*, *KIF5C*, *CEP57*, *NUSAP1*), as well as cellular components with over-represented microtubule cytoskeletons (GO:0015630) (*SEPTIN9*, *KIFC1*, *KIF5C*, *CEP57*, *GAS8*, *NUSAP1)* (Table 2).

In a separate analysis of genes that were down- and upregulated, the KEGG MAPK signaling pathway (GO:0000165) (*DUSP1*, *FOS*, *GADD45A*) and endocytosis (GO:0006897) (*PARD6B*, *KIF5C*, *MHCX1*) showed a trend toward enrichment (FDR = 0.06) with downregulated genes, while fatty acid degradation (GO:0009062) and pyruvate metabolism (GO: 0006090) (*LOC100034175*) were enriched (FDR < 0.05) with upregulated genes. The downregulated genes also enriched GO categories (FDR < 0.01) such as the cell cycle (GO:0007049) (*GPNMB*, *PARD6B*, *NUSAP1*, *ASPM*, *JUN*, *CENPF*, *KIFC1*, *GADD45A*), microtubule-based processes (GO:0007017) (*PARD6B*, *CEP57*, *KIF5C*, *NUSAP1*, *ASPM*, *KIFC1*), or terms associated with the p38MAPK cascade (GO:1900744, GO:0038066) *(DUSP1*, *GADD45A)*, whereas upregulated genes enriched (FDR < 0.05), e.g., processes connected with ATPase-coupled calcium transmembrane transporter (GO:1901894, GO:1901896) *(VMP1)* (Table S5).

The genes altered in Ex cumulus also showed a trend toward enrichment of “endocytosis” (FDR = 0.19) (*DAB2*) and “cell cycle” (FDR = 0.08) (*TTK*, *CDKN1C*) pathways.

**Figure 11:**
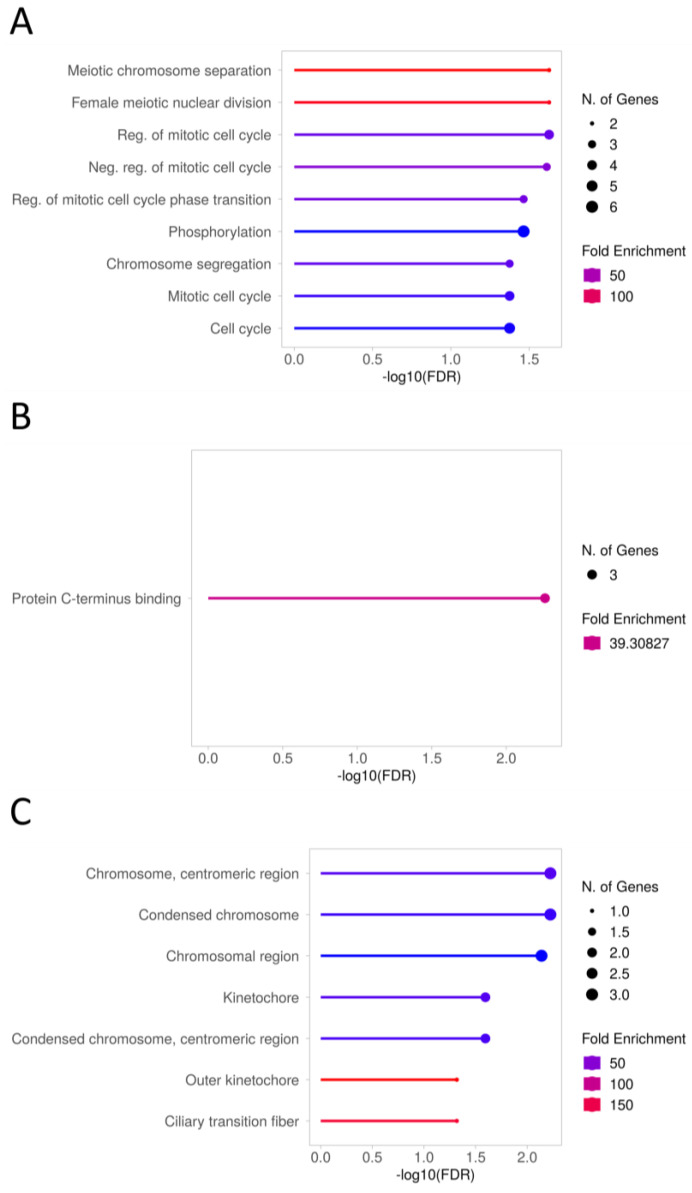
Selected biological processes (**A**), molecular functions (**B**), and cellular components (**C**) enriched in DE genes (adjP < 0.1) in CC of Ex COCs after 12 h treatment with ffEVs.

The DE genes enriched (FDR < 0.05) GO biological processes that were primarily related to the mitotic cell cycle (GO:0007346, GO:0045930, GO:1901990) (*GPNMB*, *TTK*, *CENPF*, *CDKN1C*) or chromosome segregation (GO:0007059) (*TOP2A*, *TTK*, *CENPF*). In the case of GO molecular function enrichment, statistical significance (FGR = 0.005) was reached by the term of protein c-terminus binding (GO: GO:0008022) (*TOP2A*, *DAB2*, *CENPF*), while the most enriched (FDR < 0.01) GO cellular components were associated with chromosomes (GO:0000775, GO:0000793, GO:0098687) (*TOP2A*, *TTK*, *CENPF)* (Figure 11, Table S6). The altered genes were over-represented (FDR < 0.05) with no correction for multiple testing GO-Slim biological processes in terms connected with the mitotic (GO:0045930, GO:0007346) (*TTK*, *CDKN1C)* and meiotic cell cycle (GO:1903046, GO:0051321) *(TOP2A*, *TTK)* pathways. Concerning GO-Slim molecular function, the modified genes gave an over-representation of Wnt-protein binding (GO:0017147) (*FZD5*) and phosphotransferase activity (GO:0016773) (*TTK*, *ITPKA*). The most over-represented GO-Slim cellular components, as in the case of enrichment analysis, concerned chromosomes (GO:0000776, GO:0000779, GO:0000775, GO:0098687) (*TTK*) (Table 3).

A separate examination of DE genes showed that the KEGG cell cycle (FDR = 0.09) (*TTK*) pathway and endocytosis (FRD = 0.12) (*DAB2*) tended toward enrichment with downregulated genes. Similarly, both the cell cycle (*CDKN1C*) and Wnt signaling (*FZD5*) pathway displayed a trend toward enrichment (FDR = 0.05) with upregulated genes. The downregulated genes were relevant to the overall analysis as they enriched GO categories (FDR < 0.05) associated with cell division such as meiotic chromosome separation (GO:0051307), regulation of mitotic cell cycle phase transition (GO:1901990), nuclear chromosome segregation (GO:0098813), regulation of cell cycle phase transition (GO:1901987), or chromosome segregation (GO:0007059) (*GPNMB*, *TTK*, *CENPF*, *TOP2A*). In turn, upregulated genes indicated a tendency toward enrichment (FDR = 0.05) of GO categories associated with embryonic development (GO:1903867, GO:0007350, GO:0001701, GO:0009792, GO:0001829, GO:0043009, GO:0031077) (*FZD5*), or regulation of bicellular tight junction assembly (GO:2000810) (*CDKN1C*) (Table S7).

### 2.7. qPCR Validation of RNA-Seq Results

Only 16 of the 20 samples subjected to analysis were used for qPCR validation, due to insufficient amounts of RNA. The correlation analysis between the expression levels obtained through qPCR and RNA-Seq methods indicated a moderate-to-high degree of consistency for both the *HAS2* and *DDIT4* genes. Although the correlation coefficients for individual samples exceeded 0.6 (r = 0.611 and r = 0.65, respectively), this was not statistically significant. There was a lack of consistency in the results for the *CENPF* gene obtained from both analytical methods (r = −0.26). In general, the mean expression levels of all genes assessed through qPCR had a correlation of r = 0.334 when compared to the RNA-Seq results (Table S8).

## 3. Discussion

Because ovarian follicular fluid (FF) is extremely rich in factors influencing the achievement of nuclear and cytoplasmic maturity by COCs, we used equine FF-derived EVs (ffEVs) to enrich the IVM media. We have demonstrated that small-follicle EVs are integrated into CCs and significantly support the expansion of both Cp COCs, which are known to achieve lower IVM rates, and Ex COCs, which had already started the expansion process in vivo. Additionally, we have shown that ffEVs influence the viability of the CC and also change the transcriptome and gene expression with known associations to cumulus expansion in vivo as well as the acquisition of developmental competence by the oocyte.

In recent years, studies on murine oocytes have shown that plasma-derived EVs contribute to both increased cumulus expansion and improved efficiency in reaching the MII stage during IVM [21]. These results are consistent with our results and with recent reports regarding the improvement in the IVM rate in horses [41] and in dogs [42] with the use of ffEVs. Moreover, Hung et al. [39] showed that both murine and bovine COCs supplemented with EVs derived from bovine FF also significantly increased cumulus expansion during IVM. However, this effect does not always occur, as it has been reported that porcine COCs supplemented with EVs obtained from the physiologically closer porcine FF [43] or seminal plasma [44] did not show increased expansion. Similarly, porcine ffEVs did not enhance the expansion of murine COCs [43]. This could suggest that the mechanisms responsible for cumulus expansion during oocyte maturation differ between species and, therefore, that the impact of EVs on this process as well as the quality of the composition of the EVs themselves are species-dependent.

The MAPK signaling pathway is known to be important during cumulus expansion, as well as for cytoplasmic and nuclear maturation of oocytes in vivo [45,46]. Activation of MAPK in cumulus cells is imperative for gonadotropin-induced meiotic resumption of oocytes, as it contributes to the regulation of microtubule organization and assembly of the meiotic spindle [47]. This pathway also regulates the expression of the *HAS2* and *PTGS2* genes, which are expressed in cumulus cells and are crucial for the cumulus expansion process [45,48]. Our study indicates that ffEVs influenced the expression of various genes related to the MAPK pathway (GO:0032873, GO:1900744) in the Cp COCs group, which was also characterized by a highly significant increase in expansion during IVM (*p* = 0.00006). The expression of *DUSP1* and *DDIT4* genes, which are involved in the MAPK signaling pathway, significantly decreased (padj < 0.01) in the cumulus of Cp COCs after ffEV supplementation. These genes are considered marker genes in humans, enabling the identification of oocytes defined as “pregnancy competent”, as their expression levels in the cumulus can identify good-quality oocytes for use in assisted reproductive procedures [49].

The achievement of developmental competence by oocytes involves various signaling pathways and is associated with numerous kinase proteins [50]. It has been shown that *MAP kinase p38*, over-represented by genes altered in CCs of Cp COCs (regulation of p38MAPK cascade, GO:1900744), is associated with the regulation of apoptosis in CCs in cattle [50,51]. This seems to be crucial, as during the IVM process only some CCs continue to proliferate, while others undergo spontaneous apoptosis, regardless of the medium in which maturation is performed. The cumulus survival rate may, therefore, be an important indicator of the suitability of oocytes for ART, as the degree of apoptosis is correlated with the developmental competence of oocytes in both cattle and humans [52,53]. Interestingly, in mice, MAPKs have been shown to increase the activity of *FOS*, a component of the activator protein-1 (AP-1) gene regulator, and its heterodimeric binding partner, *JUN*, in different types of cells by post-transcriptional phosphorylation [54,55]. These genes have been associated with diverse cellular alterations, such as proliferation, survival, metabolism, differentiation, steroidogenesis, prostaglandin production, and angiogenesis [56,57,58], all of which are integral for proper ovarian function, encompassing follicular development, ovulation, and luteinization [59]. Moreover, *GADD45A* has been shown to inhibit cell survival and growth [60], while *DDIT4* increases cell proliferation and reduces apoptosis [61]. In our studies, all these genes were significantly downregulated (padj < 0.05, Table 1) after ffEV supplementation in the Cp COC group, without negatively affecting CC survival. Indeed, we showed a higher survival rate (84.3%) of CCs in the supplemented Cp group compared to the control (64.7%; *p* = 0.002), and also compared to the supplemented Ex COC group (62.6%), which did not show differential expression of any of these genes. These findings could suggest that, unlike in other species, reduced expression of *FOS*, *JUN*, *GADD45A,* as well as *DDIT4* are correlated with greater cumulus survival in the domestic horse, and therefore, they may be potential marker genes for the selection of more competent COCs for assisted reproduction.

In contrast to our observations in the Cp group, we did not find that ffEVs exerted an impact on the biological processes associated with the MAPK pathway in the Ex COC group. These results align with recent findings of a notably more pronounced response of Cp COCs than Ex COCs to ffEV stimulation in enhancing nuclear maturation during the IVM process [41]. This correlation parallels the presence of MAP kinase in maturing equine oocytes, whereas it remains unphosphorylated in oocytes that are meiotically incompetent [62,63]. Supplementation with ffEVs exerted a significant influence on cumulus viability, augmenting it in the case of Cp COCs (*p* = 0.002), and reducing it for Ex COCs (*p* = 0.013), which had initiated maturation in vivo. This implies that additional stimulation with ffEVs amplifies the metabolic activity of CCs that are already in an active state in vivo (Ex COCs), potentially hastening apoptotic cell death in these cells. The heightened level of apoptosis within this particular COC group underscores the significance of ffEVs in contributing to the alteration of the biological processes associated with the negative regulation of the cell cycle (GO:0045786). This process entails the involvement of the *CDKN1C* gene, which was significantly upregulated (padj = 0.007) in the Ex COCs group. Cyclin-dependent kinase inhibitor 1C (*CDKN1C*) is a potent inhibitor of multiple G1 cyclin/Cdk complexes, and is a negative regulator of cell proliferation [64]. The inhibition of CC proliferation, linked to heightened activity of the *CDKN1C* gene, appears to be the direct catalyst for the escalated apoptosis observed in this cohort of COCs.

In addition to the effect of ffEVs on the cumulus expansion process in the Ex COC group (*p* = 0.016), we also observed an over-representation of genes related to Wnt signaling (Wnt-protein binding; GO:0017147), which is associated with embryogenesis, embryonic stem cells, and signal transduction [65]. Numerous constituents of the Wnt signaling pathway are expressed in the ovaries, COCs, and cleavage-stage mice embryos [66], and the components of the Wnt pathway play a crucial role in determining meiotic spindle orientation in a common model species—*Caenorhabditis elegans* [67]. Further, frizzled family receptors (*FZD1*, *FZD4*, and *FZD5*), which are proteins associated with the Wnt pathway, are observed during the transition from oocyte to embryo [66].

In our investigations, the *FZD5* gene was implicated in the regulation of the Wnt pathway and demonstrated slightly increased expression under the influence of ffEVs (padj = 0.0798). This observation might explain our previous findings [41] that supplementation with ffEVs contributed to the spontaneous parthenogenetic activation of Ex COCs, but not Cp COCs, during the IVM process. These outcomes also align with the current transcriptome study, where upregulated genes within this group exhibited a trend toward enrichment (FDR = 0.05) in GO categories associated with embryonic development, such as extraembryonic membrane development (GO:1903867), blastoderm segmentation (GO:0007350), and in utero (GO:0001701) or chordate embryonic development (GO:0043009). The dynamic alterations in endocytic membrane processes, constituting the principal mechanism for EV internalization, could be attributed to the transformation of phosphoinositides (PIPs) [68], present in the membranes of EVs. Hence, a plausible inference is that the presence of EVs during IVM of Ex COCs, which had commenced maturation in vivo, substantiates the sequence of events instigating parthenogenesis. The activation mechanism is presumed to be predicated on the chemical stimulation of oocytes, arising from the breakdown of PIPs within EV membranes after their internalization into the COC. This process culminates in the activation of intracellular calcium ions within the oocyte, representing a classical pathway leading to parthenogenetic activation.

Our study encountered significant limitations related to the challenges associated with working with materials of equine origin. The restricted availability of research material and low COC isolation efficiency directly influenced the scope of the investigation, resulting in a limited pool of samples for testing. Consequently, the analysis of cumulus expansion exhibited comparatively larger deviations than similar studies on other species, as the size and detached nature of the equine cumulus during IVM could have introduced a slight measurement error. Furthermore, the validation of the RNA-Seq analysis was carried out through qPCR, revealing a moderate correlation for the *DDIT4* and *HAS2* genes, while no correlation was observed for the *CENPF* gene. This lack of compliance in the results for the *CENPF* gene may be attributable to several unrecognized factors, such as the potential inadequacy of primer design leading to a failure of targeting unknown gene transcripts identified by RNA-Seq. Further, the validation process utilized a minimal quantity of RNA matrix, and some samples had RNA concentrations below the recommended threshold for qPCR, suggesting suboptimal conditions for this method. An insufficient amount of starting material led to the exclusion of several samples, resulting in a limited pool for qPCR validation and potentially influencing the overall correlation between the two methods and its significance. Despite these challenges, the overall correlation of 0.334 (and r > 0.6 for two of the three genes analyzed) indicates no significant bias in the relative quantification of gene expression by the RNA-Seq method.

All these findings suggest that ffEV addition affects cumulus expansion and viability and the expression of several genes associated with oocyte and cumulus function, and thus can modulate the efficacy of COCs in assisted reproduction techniques, via the accomplishment of meiotic maturation in oocytes as well as by fostering cytoplasmic maturity and subsequent developmental competency. However, the functions of EVs mirror the conditions in the source microenvironment, influencing various molecular and phenotypic alterations in both cumulus and granulosa cells. It was for this reason that we decided to focus on small ovarian follicles, which seem to be a richer source of EVs that are also more easily internalized by CCs [39], and show a greater ability to induce CC proliferation [40] compared to EVs from medium and large follicles. In future research, it could be of value to extend the scope to include follicles of varying sizes, thereby ensuring a more comprehensive understanding of the effects of ffEVs. In addition, the inclusion of follicles from different phases of the estrous cycle could also provide valuable insights.

## 4. Materials and Methods

### 4.1. Experimental Design

For cumulus viability and expansion, selected COCs were cultured in IVM medium for 38 h with ffEV supplementation (200 μg protein/mL in DPBS, corresponding to 6 × 10^9^ EVs particles/mL), according to Hung et al. [39] and our recent study [41], or in medium alone (control). For transcriptome analysis, COCs were matured for 12 h, after which cumulus cells were removed and frozen until RNA isolation.

COCs were randomly assigned to the following groups:

- Compacted (Cp) control;
- Cp + ffEVs;
- Expanded (Ex) control;
- Ex + ffEVs.

### 4.2. Source of Ovaries

The study material consisted of ovaries obtained from 43 adult mares of unrecorded age and breed. The mares were slaughtered for purposes unrelated to the study. Ovaries were collected within 15 min of slaughter and placed in a thermal box, covered with sterile gauze, and transported to the laboratory at room temperature (RT, ~22 °C) within 2 h.

### 4.3. Follicular Fluid and COC Collection

Follicular fluid (FF) was aspirated from small (<20 mm) equine ovarian follicles using a 12-gauge needle. Four independent collections of FF were conducted over a 4-month period. FF from each collection was pooled before EVs extraction. Cumulus oocyte complexes (COCs) were recovered using the curettage method as previously described [41] and classified based on cumulus morphology. Compact COCs (Cp) included oocytes with compacted CCs or with minor signs of expansion in the outer cells; expanded COCs (Ex) included oocytes with signs of expansion in more than two-thirds of the surrounding CCs, and corona radiata (Cr) oocytes surrounded by the innermost layers of CCs. Only Cp and Ex COCs were used in this study. Directly after collection, all COCs were placed in holding medium (EQ-Hold, IVF-Bioscience, Falmouth, UK) at RT.

### 4.4. In Vitro Maturation of Oocyte (IVM)

Selected COCs were grouped as previously described and placed into a 4-well dish in 400 μL of commercial maturation medium (EQ-IVM, IVF-Bioscience, Falmouth, UK), either alone or with ffEVs. All COCs were cultured at 38.2 °C under a humidified atmosphere of 6% CO_2_ in air.

### 4.5. Extracellular Vesicle (EV) Extraction

Extracellular vesicles were extracted using a differential ultracentrifugation method, as previously described [41]. Briefly, after aspiration, FF was diluted with Dulbecco’s Phosphate Buffered Saline (DPBS) (Sigma-Aldrich, Merck KGaA, Darmstadt, Germany) (1:1 *v*/*v*) and immediately centrifuged at 700× *g* for 10 min, 2000× *g* for 10 min to eliminate residual cumulus cells and oocytes, and 12,000× *g* for 30 min to remove cell debris and large particles. Centrifuged FF was filtered through a 0.22 µm filter and centrifuged twice by ultracentrifugation at 120,000× *g* for 70 min. All centrifugations were performed at 4 °C. The supernatant was carefully removed and the pellets, comprised of ffEVs, were suspended in DPBS to a final concentration of approximately 6 × 10^11^ particles/mL and 20 mg protein/mL and stored at −80 °C. The concentration of EVs was measured by quantifying the protein content using a Pierce Coomassie Plus (Bradford, UK) Assay Kit (Thermo Fisher, Waltham, MA, USA) as per the manufacturer’s protocol.

### 4.6. Transmission Electron Microscopy (TEM)

The morphological characterization of ffEVs was made using TEM. Briefly, 3 µL droplets of purified EVs were placed on formvar/carbon-coated 200 mesh grids (Agar Scientific, Stansted, UK). The droplets were allowed to adsorb onto the grid for 15 min, after which the remaining liquid was drained. To create the contrast, the grids were treated with 2% uranyl acetate water solution (Polysciences, Warrington, PA, USA) for 2 min and then air-dried. Samples were visualized with a JEM 1400 TEM (JEOL Ltd., Tokyo, Japan) at 80 kV and the high-resolution digital images of ffEVs were captured using a digital camera (Morada TEM CCD camera, Olympus, Hamburg, Germany).

### 4.7. Nanoparticle Tracking Analysis (NTA)

Particle concentration and size distribution in ffEV samples were assessed by NanoSight NS300 3.4 Build 3.4.003 analytical software (Malvern Instruments, Malvern, UK) employing nanoparticle tracking analysis (NTA). Follicular EV preparations were diluted before analysis in 0.22 µm filtered DPBS without Ca^2+^ or Mg^2+^ (Lonza, Basel, Switzerland). Each sample underwent three 1 min recordings with an sCMOS camera level set at 13. The recordings were conducted at a consistent temperature of 20 °C, producing three histograms for each sample, which were then averaged. The D90 parameter, where 90% of the EV population had a diameter equal to or less than the reported mean value, was also determined.

### 4.8. Flow Cytometry (FC)

High-resolution FC was used for the detection of EV markers, as previously described [41]. Briefly, ffEVs were stained with APC-conjugated antibodies against the markers CD63 (clone MEM-259; Thermo Fisher Scientific, Waltham, MA, USA) and CD81 (clone 5A6; BioLegend, San Diego, CA, USA), or the appropriate isotype control (mouse IgG1 k APC, Miltenyi Biotec, Bergisch Gladbach, Germany). Prior to staining, antibodies were reconstituted in DPBS without Ca^2+^ or Mg^2+^ (Lonza, Basel, Switzerland), centrifuged at 21,000× *g* for 20 min at 4 °C to eliminate potential protein aggregates, and the supernatants transferred to fresh tubes. For the staining process, ffEV samples were introduced to the supernatants and incubated for 20 min at 4 °C. Analysis was conducted using an Apogee A60-Micro-PLUS cytometer (Apogee Flow Systems, Hemel Hempstead, UK) and Histogram software v242 (Apogee Flow Systems) was used to determine the percentage of positive events within the gated population.

### 4.9. ffEV Labeling and Uptake by COCs

EVs were labeled using an ExoGlow-protein EV labeling kit (System Biosciences, Palo Alto, CA, USA) as per the manufacturer’s protocol. Briefly, ffEVs were incubated for 20 min with the labeling dye at 37 °C to induce vesicular protein conjugation with the dye molecules. The solution was incubated for 2 h at 4 °C with the ExoQuick-TC solution, followed by centrifugation at 10,000× *g* for 10 min to remove unlabeled reagent molecules. For a negative control, sterile PBS was incubated with labeling dye and treated in the same manner. Labeled ffEV pellets were dissolved in DPBS and their concentration was measured with a Pierce Coomassie Plus (Bradford) Assay Kit (Thermo Fisher Scientific, Waltham, MA, USA). EVs were added to COC cultures (200 μg/mL) and the corresponding volume of prepared mixture was added for the negative control. After IVM, all COCs were fixed in buffered formalin (4 °C) for 24–48 h and stained for 10 min with Alexa Fluor™ 568 Phalloidin (Invitrogen™, Thermo Fisher Scientific, Waltham, MA, USA) for actin filament staining, then placed on a glass slide covered with mounting medium (9:1 glycerol/PBS) containing 2.5 μg/mL Hoechst 33342 (Sigma-Aldrich, Merck KGaA, Darmstadt, Germany) for nuclei visualization. Serial z-sections were imaged (1-μm thickness) on a Leica SP8 WRL scanning confocal microscope with a 63× objective.

### 4.10. Cumulus Expansion Assessment

All COCs were imaged by OptaView 7.1 software on a Nikon Eclipse E600 inverted microscope immediately after transfer to a culture dish (0 h) and following IVM (38 h). Images were captured using a Nikon E Plan 10×/0.25 WD 12.5 objective and an Opta-Tech camera. The size of each COC was measured using ImageJ software v1.8.0. For each COC, two intersecting perpendicular dimensions were measured and then averaged, resulting in an average measurement (µm). COCs were cultured in groups of 8–14 in five different replicates. The overall measure of expansion was the average change in dimensions from 0 h to 38 h for all replicates.

### 4.11. Cumulus Viability Assessment

All COCs were placed on a glass slide in a drop of DPBS containing 1 mg/mL ethidium bromide (EtBr) and 0.015 mg/mL fluorescein diacetate (FDA), according to Nowak et al. [69], but with concentration modifications. The evaluation was performed with the use of a Nikon Eclipse E600 fluorescence microscope supplied with an appropriate filter. Cells showing green fluorescence were classified as live, while those that demonstrated red-orange fluorescence were recognized as dead. Microphotographs were taken using an Opta-Tech camera and OptaView 7.1 software. The surface areas of the dead and live cells were estimated using ImageJ software. Cumulus viability was calculated as the ratio of live to dead cells [70].

### 4.12. RNA Isolation

After 12 h of IVM, COCs were transferred to the washing medium, and cumulus cells were harvested using small glass pipettes and frozen at −80 °C until RNA isolation. The cumulus cells from each replicate (sample) were used for total RNA isolation using a modified TRIzol™ reagent protocol designed for oocyte RNA isolation and low material input [71,72]. Briefly, 100 µL of TRI Reagent (Thermo Fisher Scientific, Waltham, MA, USA) was added to thawed cumulus cells, incubated at RT, and centrifuged at 12,000× *g* for 10 min (4 °C). After that, 50 µL chloroform was added, incubated at RT, and centrifuged at 12,000× *g* for 15 min (4 °C). The aqueous phase was transferred to a fresh tube and incubated for 10 min with 2-propanol, followed by centrifugation at 12,000× *g* for 10 min. The supernatant was removed, and the RNA was washed with 75% EtOH and suspended in ultra-pure water. The isolated RNA was quality-controlled (TapeStation System—RIN) and quantified using a Qubit fluorometer (Qubit™ RNA HS Assay Kit, Thermo Fisher Scientific, Waltham, MA, USA), and used for the sequencing library preparation.

### 4.13. 3′ mRNA-Seq Library Preparation and Sequencing

Libraries were prepared according to the manufacturer’s protocol (QuantSeq 3′ mRNA Library Prep Kit for Illumina, Lexogen GmbH, Vienna, Austria). In brief, the process of library generation was initiated by oligo-dT priming. First-strand synthesis and RNA removal were followed by random-primed synthesis of the complementary strand (second-strand synthesis). Illumina-specific linker sequences were also introduced by the primers. The resulting double-stranded cDNA was purified with magnetic beads (AMPure XP, Beckman Coulter, Brea, CA, USA). PCR amplification of the library introduced the complete adapter sequences required for cluster generation and sample multiplexing. Prepared Illumina libraries were quantified (Qubit dsDNA fluorometric assay), quality-controlled (Agilent TapeStation D1000 tape and reagents), normalized, multiplexed, and finally sequenced in a single-end 75 bp run on the Illumina System to obtain about 5–8 M SE reads per sample.

### 4.14. Transcriptome Analysis and Data Analysis

Data analysis of the sequencing results involved reads quality control (FastQC software v0.11.9), filtering (Flexbar) [73], and mapping against a reference genome with STAR aligner software v2.7.10a [74]. The reference genome used was EquCab3.0 with v107 ENSEMBL gene annotation [75]. Reads counting was performed using Htseq-count software v3.1. Read counts were further processed using the differential expression (DE) analysis pipeline with DESeq2 software v1.42.1 [76]. Genes were considered as DE when the FDR (false discovery rate) was <0.1, unless otherwise stated. The DE genes were analyzed for their function and biological significance using over-representation tests in gene ontology (GO) and KEEG pathways using PANTHER18.0 software [77]. Additional gene set enrichment analysis was also performed with ShinyGO 0.77 [78] and KOBAS3.0 software [79]. All over-representation and enrichment tests were conducted in relation to all identified *Equus caballus* genes, unless stated otherwise. Expression profile differentiation was additionally visualized using PCA, MA, volcano, and correlation plots.

### 4.15. RT-PCR and qPCR

The results obtained from sequencing were validated using the quantitative real-time PCR (q-PCR) method. First, reverse transcription PCR (RT-PCR) was performed using 0.1 μg of the total RNA that remained following the RNA-Seq analysis for each sample using a High-Capacity cDNA Reverse Transcription Kit (Applied Biosystems, Thermo Fisher Scientific, Waltham, MA, USA) according to the manufacturer’s protocol. Briefly, the estimation of the expression of one gene that promotes cumulus expansion and oocyte maturation (*HAS2*), one gene that regulates chromosome segregation (*CENPF*), and one DNA damage gene (*DDIT4*) was performed using PowerUp™ SYBR™ Green Master Mix for qPCR (Applied Biosystems, Thermo Fisher Scientific, Waltham, MA, USA) as recommended by the manufacturer. Gene expression was assessed using the QuantStudio™ 3 Real-Time PCR System (Applied Biosystems, Thermo Fisher Scientific, Waltham, MA, USA). Target gene expression levels were normalized to glyceraldehyde-3-phosphate dehydrogenase (*GAPDH*) and tyrosine 3-monooxygenase/tryptophan 5-monooxygenase activation protein zeta (*YWHAZ*) using the geometric average of the two reference genes selected as stable genes (M < 1.5) by geNorm v3.4 [80]. Target and housekeeping gene primers were designed with Primer Express™ Software v3.0.1 (Applied Biosystems, Thermo Fisher Scientific, Waltham, MA, USA) and were supplied by Genomed (Table S9).

### 4.16. Statistical Analysis

Statistical data analysis was performed using Statistica 13.0 (StatSoftland) software. The normality of distributions was examined using the Kolmogorov–Smirnov test. Cumulus expansion and viability in the presence or absence of ffEVs were compared by one-way ANOVA. Data in charts are presented as mean or percentages ± standard deviation and statistical significance was considered at *p* < 0.05 unless otherwise stated. The confocal microscopy images underwent a qualitative analysis, with a comparison to the negative control group. The individual gene expression levels were calculated by relative quantitative (RQ) analysis and the Pfaffl model, which included reaction efficiency for individual genes [81]. An assessment of the correlation coefficient between the gene expression levels identified by both RNA-Seq and qPCR methods was conducted. This analysis was performed for each gene within the sampled data, with particular emphasis on the averaged expression values.

## Figures and Tables

**Table 1:** Differentially expressed genes between control and ffEVs supplementation group in Cp and Ex COCs (padj < 0.1).

COC	Gene Symbol	Gene	Description	Log2fold	Padj
Cp	ENSECAG00000013822	*KIF5C*	kinesin family member 5C	−1.82667	9.44 × 10^−5^
	ENSECAG00000024531	*ADM*	adrenomedullin	−1.85264	9.44 × 10^−5^
	ENSECAG00000009958	*DUSP1*	dual specificity phosphatase 1	−1.24247	9.53 × 10^−5^
	ENSECAG00000020042	*GAS8*	growth arrest specific 8	5.207698	0.002822
	ENSECAG00000014260	*FOS*	Fos proto-onco, AP-1 transcription factor subunit	−1.43215	0.006363
	ENSECAG00000016495	*NUSAP1*	nucleolar and spindle-associated protein 1	−1.36047	0.006363
	ENSECAG00000004107	*DDIT4*	DNA damage-inducible transcript 4	−1.32112	0.006983
	ENSECAG00000024055	*JUN*	Jun proto-oncogene, AP-1 transcription factor subunit	−1.06773	0.011582
	ENSECAG00000003112	*protein coding*	uncharacterized	5.77611	0.012615
	ENSECAG00000048140	*lncRNA*	uncharacterized	6.058224	0.012615
	ENSECAG00000010132	*HMGB2*	high mobility group box 2	−1.19162	0.01456
	ENSECAG00000049390	*protein coding*	uncharacterized	6.568594	0.01456
	ENSECAG00000033482	*MAF*	MAF bZIP transcription factor	−2.62299	0.015728
	ENSECAG00000034891	*FOXL2*	forkhead box L2	−0.88989	0.019069
	ENSECAG00000046492	*lncRNA*	uncharacterized	5.316257	0.019069
	ENSECAG00000040631	*GADD45A*	growth arrest and DNA damage-inducible alpha	−1.38945	0.025639
	ENSECAG00000009294	*PARD6B*	par-6 family cell polarity regulator beta	−1.37246	0.028419
	ENSECAG00000057832	*lncRNA*	uncharacterized	5.798079	0.039461
	ENSECAG00000019564	*protein coding*	uncharacterized	5.979852	0.043859
	ENSECAG00000024127	*CENPF*	centromere protein F	−1.20947	0.043859
	ENSECAG00000049668	*lncRNA*	uncharacterized	6.15095	0.050595
	ENSECAG00000019267	*protein coding*	uncharacterized	5.56764	0.050842
	ENSECAG00000004339	*MIS18BP1*	MIS18 binding protein 1	−1.44315	0.050887
	ENSECAG00000058688	*lncRNA*	uncharacterized	1.808977	0.05315
	ENSECAG00000048591	*lncRNA*	uncharacterized	−5.84793	0.053781
	ENSECAG00000010938	*CEP57*	centrosomal protein 57	−0.80552	0.056617
	ENSECAG00000020246	*SEPTIN9*	septin 9	0.740021	0.056617
	ENSECAG00000034339	*MHCX1*	MHC class I heavy chain	−1.121	0.056617
	ENSECAG00000020645	*ASPM*	assembly factor for spindle microtubules	−1.31423	0.061879
	ENSECAG00000005570	*VMP1*	vacuole membrane protein 1	0.634005	0.082138
	ENSECAG00000006404	*SLC17A3*	solute carrier family 17 member 3	5.861373	0.082138
	ENSECAG00000010064	*OLFM3*	olfactomedin 3	5.769174	0.082138
	ENSECAG00000024196	*KIFC1*	kinesin family member C1	−1.22762	0.082138
	ENSECAG00000047902	*lncRNA*	uncharacterized	5.046975	0.082138
	ENSECAG00000000658	*GPNMB*	glycoprotein nmb	−5.85274	0.086203
	ENSECAG00000010303	*CIAO2A*	cytosolic iron–sulfur assembly component 2A	3.922212	0.097695
Ex	ENSECAG00000009081	*KIAA1210*	KIAA1210	−1.54446	0.000302
	ENSECAG00000031389	*CDKN1C*	cyclin-dependent kinase inhibitor 1C	1.240626	0.0074
	ENSECAG00000024127	*CENPF*	centromere protein F	−1.15973	0.010131
	ENSECAG00000000658	*GPNMB*	glycoprotein nmb	−2.82836	0.068233
	ENSECAG00000011581	*protein coding*	uncharacterized	1.20007	0.068233
	ENSECAG00000007309	*FZD5*	frizzled class receptor 5	0.685669	0.079819
	ENSECAG00000057237	*lncRNA*	uncharacterized	−2.32694	0.090332
	ENSECAG00000029287	*HOPX*	HOP homeobox	1.480728	0.094982
	ENSECAG00000007607	*PTPRG*	protein tyrosine phosphatase receptor type G	−0.6582	0.097187
	ENSECAG00000013053	*TOP2A*	DNA topoisomerase II alpha	−1.05374	0.097187
	ENSECAG00000013597	*DAB2*	DAB adaptor protein 2	−1.01644	0.097187
	ENSECAG00000014517	*CDCA3*	cell division cycle-associated 3	−1.59657	0.097187
	ENSECAG00000018981	*ITPKA*	inositol-trisphosphate 3-kinase A	−0.76345	0.097187
	ENSECAG00000019296	*GUF1*	GTP binding elongation factor GUF1	1.778967	0.097187
	ENSECAG00000023776	*TTK*	TTK protein kinase	−3.71007	0.097187
	ENSECAG00000044783	*PCDHGA7*	protocadherin gamma subfamily A, 7	−3.8215	0.097187

**Table 2:** Selected biological processes, molecular functions, and cellular components over-represented in DE genes (adjP < 0.1) in CCs of Cp COCs after 12 h treatment with ffEVs (PANTHER18.0). GO—gene ontology term; P—adjusted *p*-value.

GO	Description	Term ID	P	Engaged Genes
Biological processes	microtubule-based process	GO:0007017	1.34 × 10^−3^	*KIFC1*, *KIF5C*, *PARD6B*, *NUSAP1*
	cytoskeleton-dependent cytokinesis	GO:0061640	1.47 × 10^−3^	*SEPTIN9*, *NUSAP1*
	cytokinesis	GO:0000910	1.54 × 10^−3^	*SEPTIN9*, *NUSAP1*
	cell division	GO:0051301	2.21 × 10^−3^	*SEPTIN9*, *NUSAP1*
	negative regulation of stress-activated MAPK cascade	GO:0032873	5.16 × 10^−3^	*DUSP1*
	regulation of p38MAPK cascade	GO:1900744	5.16 × 10^−3^	*DUSP1*
	negative regulation of intracellular signal transduction	GO:1902532	5.38 × 10^−3^	*DUSP1*, *DDIT4*
	establishment of organelle localization	GO:0051656	7.51 × 10^−3^	*KIF5C*, *NUSAP1*
	cell cycle process	GO:0022402	7.80 × 10^−3^	*SEPTIN9*, *PARD6B*, *NUSAP1*
	endoderm development	GO:0007492	9.01 × 10^−3^	*DUSP1*
Molecular functions	microtubule binding	GO:0008017	4.48 × 10^−5^	*KIFC1*, *KIF5C*, *CEP57*, *NUSAP1*
	tubulin binding	GO:0015631	9.48 × 10^−5^	*KIFC1*, *KIF5C*, *CEP57*, *NUSAP1*
	microtubule motor activity	GO:0003777	2.46 × 10^−3^	*KIFC1*, *KIF5C*
	cytoskeletal protein binding	GO:0008092	2.77 × 10^−3^	*KIFC1*, *KIF5C*, *CEP57*, *NUSAP1*
	ATP hydrolysis activity	GO:0016887	3.48 × 10^−3^	*KIFC1*, *KIF5C*
	ribonucleoside triphosphate phosphatase activity	GO:0017111	3.79 × 10^−3^	*SEPTIN9*, *KIFC1*, *KIF5C*
	efflux transmembrane transporter activity	GO:0015562	3.87 × 10^−3^	*SLC17A3*, *ABCG2*
	xenobiotic transmembrane transporter activity	GO:0042910	5.16 × 10^−3^	*ABCC1*, *SLC17A3*, *SLC6A6*
	pyrophosphatase activity	GO:0016462	5.44 × 10^−3^	*SEPTIN9*, *KIFC1*, *KIF5C*
	hydrolase activity, acting on acid anhydrides, in phosphorus-containing anhydrides	GO:0016818	5.49 × 10^−3^	*SEPTIN9*, *KIFC1*, *KIF5C*
Cellular components	microtubule cytoskeleton	GO:0015630	1.69 × 10^−5^	*SEPTIN9*, *KIFC1*, *KIF5C*, *CEP57*, *GAS8*, *NUSAP1*
	cytoskeleton	GO:0005856	3.80 × 10^−4^	*SEPTIN9*, *KIFC1*, *KIF5C*, *CEP57*, *GAS8*, *NUSAP1*
	microtubule	GO:0005874	9.53 × 10^−4^	*KIFC1*, *KIF5C*, *GAS8*
	intracellular non-membrane-bounded organelle	GO:0043232	2.59 × 10^−3^	*SEPTIN9*, *KIFC1*, *MIS18BP1*, *KIF5C*, *CEP57*, *GAS8*, *NUSAP1*
	non-membrane-bounded organelle	GO:0043228	2.59 × 10^−3^	*SEPTIN9*, *KIFC1*, *MIS18BP1*, *KIF5C*, *CEP57*, *GAS8*, *NUSAP1*
	polymeric cytoskeletal fiber	GO:0099513	3.79 × 10^−3^	*KIFC1*, *KIF5C*, *GAS8*
	microtubule associated complex	GO:0005875	6.94 × 10^−3^	*KIFC1*, *KIF5C*
	supramolecular fiber	GO:0099512	8.14 × 10^−3^	*KIFC1*, *KIF5C*, *GAS8*
	supramolecular polymer	GO:0099081	8.21 × 10^−3^	*KIFC1*, *KIF5C*, *GAS8*
	cell cortex	GO:0005938	9.00 × 10^−3^	*SEPTIN9*, *PARD6B*

**Table 3:** Selected biological processes, molecular functions, and cellular components over-represented in DE genes (adjP < 0.1) in CC of Ex COCs after 12 h treatment with ffEVs (PANTHER18.0). GO—gene ontology term; P—adjusted *p*-value.

GO	Description	Term ID	P	Engaged Genes
Biological processes	negative regulation of mitotic cell cycle	GO:0045930	5.86 × 10^−4^	*TTK*, *CDKN1C*
	meiotic cell cycle process	GO:1903046	7.47 × 10^−4^	*TOP2A*, *TTK*
	meiotic cell cycle	GO:0051321	9.55 × 10^−4^	*TOP2A*, *TTK*
	negative regulation of cell cycle	GO:0045786	1.34 × 10^−3^	*TTK*, *CDKN1C*
	regulation of mitotic cell cycle	GO:0007346	2.03 × 10^−3^	*TTK*, *CDKN1C*
	chromosome segregation	GO:0007059	2.53 × 10^−3^	*TOP2A*, *TTK*
	protein localization to kinetochore	GO:0034501	4.02 × 10^−3^	*TTK*
	sexual reproduction	GO:0019953	5.82 × 10^−3^	*TOP2A*, *TTK*
	positive regulation of endocytosis	GO:0045807	6.02 × 10^−3^	*DAB2*
	negative regulation of mitotic sister chromatid segregation	GO:0033048	7.35 × 10^−3^	*TTK*
Molecular functions	Wnt-protein binding	GO:0017147	1.80 × 10^−2^	*FZD5*
	ribosome binding	GO:0043022	2.13 × 10^−2^	*GUF1*
	ribonucleoprotein complex binding	GO:0043021	3.04 × 10^−2^	*GUF1*
	integrin binding	GO:0005178	3.50 × 10^−2^	*GPNMB*
	protein tyrosine phosphatase activity	GO:0004725	4.66 × 10^−2^	*PTPRG*
	phosphotransferase activity, alcohol group as acceptor	GO:0016773	4.69 × 10^−2^	*TTK*, *ITPKA*
	protein-containing complex binding	GO:0044877	4.82 × 10^−2^	*GUF1*, *GPNMB*
Cellular components	kinetochore	GO:0000776	2.45 × 10^−2^	*TTK*
	condensed chromosome, centromeric region	GO:0000779	2.58 × 10^−2^	*TTK*
	chromosome, centromeric region	GO:0000775	3.24 × 10^−2^	*TTK*
	chromosomal region	GO:0098687	3.69 × 10^−2^	*TTK*

## Data Availability

The original contributions presented in the study are included in the article/Supplementary Materials; further inquiries can be directed to the corresponding author.

## Supplementary Materials

The following supporting information can be downloaded at: https://www.mdpi.com/1422-0067/25/6/3262/s1.

## References

[1] Reader K., Stanton J.-A., Juengel J. (2017). The Role of Oocyte Organelles in Determining Developmental Competence. Biology.

[2] Sirard M.-A., Richard F., Blondin P., Robert C. (2006). Contribution of the Oocyte to Embryo Quality. Theriogenology.

[3] Eppig J.J. (1979). FSH Stimulates Hyaluronic Acid Synthesis by Oocyte–Cumulus Cell Complexes from Mouse Preovulatory Follicles. Nature.

[4] Zhou C.-J., Wu S.-N., Shen J.-P., Wang D.-H., Kong X.-W., Lu A., Li Y.-J., Zhou H.-X., Zhao Y.-F., Liang C.-G. (2016). The Beneficial Effects of Cumulus Cells and Oocyte-Cumulus Cell Gap Junctions Depends on Oocyte Maturation and Fertilization Methods in Mice. PeerJ.

[5] Pangas S.A., Matzuk M.M. (2005). The Art and Artifact of GDF9 Activity: Cumulus Expansion and the Cumulus Expansion-Enabling Factor1. Biol. Reprod..

[6] Fülöp C., Salustri A., Hascall V.C. (1997). Coding Sequence of a Hyaluronan Synthase Homologue Expressed during Expansion of the Mouse Cumulus–Oocyte Complex. Arch. Biochem. Biophys..

[7] Davis B.J., Lennard D.E., Lee C.A., Tiano H.F., Morham S.G., Wetsel W.C., Langenbach R. (1999). Anovulation in Cyclooxygenase-2-Deficient Mice Is Restored by Prostaglandin E2 and Interleukin-1β. Endocrinology.

[8] Marei W.F., Abayasekara D.R.E., Wathes D.C., Fouladi-Nashta A.A. (2014). Role of PTGS2-Generated PGE2 during Gonadotrophin-Induced Bovine Oocyte Maturation and Cumulus Cell Expansion. Reprod. Biomed. Online.

[9] Chen L., Russell P.T., Larsen W.J. (1993). Functional Significance of Cumulus Expansion in the Mouse: Roles for the Preovulatory Synthesis of Hyaluronic Acid within the Cumulus Mass. Mol. Reprod. Dev..

[10] Hess K.A., Chen L., Larsen W.J. (1999). Inter-α-Inhibitor Binding to Hyaluronan in the Cumulus Extracellular Matrix Is Required for Optimal Ovulation and Development of Mouse Oocytes1. Biol. Reprod..

[11] Rodgers R.J., Irving-Rodgers H.F. (2010). Formation of the Ovarian Follicular Antrum and Follicular Fluid. Biol. Reprod..

[12] Nandi S., Kumar V.G., Manjunatha B.M., Gupta P.S.P. (2007). Biochemical Composition of Ovine Follicular Fluid in Relation to Follicle Size. Dev. Growth Differ..

[13] Gosden R.G., Hunter R.H.F., Telfer E., Torrance C., Brown N. (1988). Physiological Factors Underlying the Formation of Ovarian Follicular Fluid. Reproduction.

[14] McNatty K.P., Jones R.E. (1978). Follicular Fluid. The Vertebrate Ovary.

[15] Espey L.L., Lipner H., Knobil E., Neill J.D. (1994). Ovulation. The Physiology of Reproduction.

[16] Ambekar A.S., Nirujogi R.S., Srikanth S.M., Chavan S., Kelkar D.S., Hinduja I., Zaveri K., Prasad T.S.K., Harsha H.C., Pandey A. (2013). Proteomic Analysis of Human Follicular Fluid: A New Perspective towards Understanding Folliculogenesis. J. Proteom..

[17] Battaglia R., Musumeci P., Ragusa M., Barbagallo D., Scalia M., Zimbone M., Lo Faro J.M., Borzì P., Scollo P., Purrello M. (2020). Ovarian Aging Increases Small Extracellular Vesicle CD81+ Release in Human Follicular Fluid and Influences MiRNA Profiles. Aging.

[18] da Silveira J.C., Andrade G.M., Simas R.C., Martins-Júnior H.A., Eberlin M.N., Smith L.C., Perecin F., Meirelles F.V. (2021). Lipid Profile of Extracellular Vesicles and Their Relationship with Bovine Oocyte Developmental Competence: New Players in Intra Follicular Cell Communication. Theriogenology.

[19] da Silveira J.C., Veeramachaneni D.N.R., Winger Q.A., Carnevale E.M., Bouma G.J. (2012). Cell-Secreted Vesicles in Equine Ovarian Follicular Fluid Contain MiRNAs and Proteins: A Possible New Form of Cell Communication Within the Ovarian Follicle. Biol. Reprod..

[20] Mobarak H., Heidarpour M., Lolicato F., Nouri M., Rahbarghazi R., Mahdipour M. (2019). Physiological Impact of Extracellular Vesicles on Female Reproductive System; Highlights to Possible Restorative Effects on Female Age-related Fertility. BioFactors.

[21] Javadi M., Soleimani Rad J., Pashaiasl M., Farashah M.S.G., Roshangar L. (2022). The Effects of Plasma-Derived Extracellular Vesicles on Cumulus Expansion and Oocyte Maturation in Mice. Reprod. Biol..

[22] Chen Y., Li G., Liu M.-L. (2018). Microvesicles as Emerging Biomarkers and Therapeutic Targets in Cardiometabolic Diseases. Genom. Proteom. Bioinform..

[23] Mashouri L., Yousefi H., Aref A.R., Ahadi A.m., Molaei F., Alahari S.K. (2019). Exosomes: Composition, Biogenesis, and Mechanisms in Cancer Metastasis and Drug Resistance. Mol. Cancer.

[24] Kakarla R., Hur J., Kim Y.J., Kim J., Chwae Y.-J. (2020). Apoptotic Cell-Derived Exosomes: Messages from Dying Cells. Exp. Mol. Med..

[25] Théry C., Witwer K.W., Aikawa E., Alcaraz M.J., Anderson J.D., Andriantsitohaina R., Antoniou A., Arab T., Archer F., Atkin-Smith G.K. (2018). Minimal Information for Studies of Extracellular Vesicles 2018 (MISEV2018): A Position Statement of the International Society for Extracellular Vesicles and Update of the MISEV2014 Guidelines. J. Extracell. Vesicles.

[26] Lim L.P., Lau N.C., Garrett-Engele P., Grimson A., Schelter J.M., Castle J., Bartel D.P., Linsley P.S., Johnson J.M. (2005). Microarray Analysis Shows That Some MicroRNAs Downregulate Large Numbers of Target MRNAs. Nature.

[27] Sætrom P., Snøve O., Rossi J.J. (2007). Epigenetics and MicroRNAs. Pediatr. Res..

[28] Lei L., Jin S., Gonzalez G., Behringer R.R., Woodruff T.K. (2010). The Regulatory Role of Dicer in Folliculogenesis in Mice. Mol. Cell Endocrinol..

[29] Santonocito M., Vento M., Guglielmino M.R., Battaglia R., Wahlgren J., Ragusa M., Barbagallo D., Borzì P., Rizzari S., Maugeri M. (2014). Molecular Characterization of Exosomes and Their MicroRNA Cargo in Human Follicular Fluid: Bioinformatic Analysis Reveals That Exosomal MicroRNAs Control Pathways Involved in Follicular Maturation. Fertil. Steril..

[30] da Silveira J.C., Winger Q.A., Bouma G.J., Carnevale E.M. (2015). Effects of Age on Follicular Fluid Exosomal MicroRNAs and Granulosa Cell Transforming Growth Factor-β Signalling during Follicle Development in the Mare. Reprod. Fertil. Dev..

[31] da Silveira J.C., de Andrade G.M., Nogueira M.F.G., Meirelles F.V., Perecin F. (2015). Involvement of MiRNAs and Cell-Secreted Vesicles in Mammalian Ovarian Antral Follicle Development. Reprod. Sci..

[32] Sohel M.d.M.H., Hoelker M., Noferesti S.S., Salilew-Wondim D., Tholen E., Looft C., Rings F., Uddin M.J., Spencer T.E., Schellander K. (2013). Exosomal and Non-Exosomal Transport of Extra-Cellular MicroRNAs in Follicular Fluid: Implications for Bovine Oocyte Developmental Competence. PLoS ONE.

[33] de Ávila A.C.F.C.M., da Silveira J.C. (2020). Role of Extracellular Vesicles during Oocyte Maturation and Early Embryo Development. Reprod. Fertil. Dev..

[34] Czernek L., Düchler M. (2020). Exosomes as Messengers between Mother and Fetus in Pregnancy. Int. J. Mol. Sci..

[35] Hasan M.M., Viil J., Lättekivi F., Ord J., Reshi Q.U.A., Jääger K., Velthut-Meikas A., Andronowska A., Jaakma Ü., Salumets A. (2020). Bovine Follicular Fluid and Extracellular Vesicles Derived from Follicular Fluid Alter the Bovine Oviductal Epithelial Cells Transcriptome. Int. J. Mol. Sci..

[36] de Alcântara-Neto A.S., Cuello C., Uzbekov R., Bauersachs S., Mermillod P., Almiñana C. (2022). Oviductal Extracellular Vesicles Enhance Porcine In Vitro Embryo Development by Modulating the Embryonic Transcriptome. Biomolecules.

[37] Bauersachs S., Mermillod P., Almiñana C. (2020). The Oviductal Extracellular Vesicles’ RNA Cargo Regulates the Bovine Embryonic Transcriptome. Int. J. Mol. Sci..

[38] da Silveira J.C., Carnevale E.M., Winger Q.A., Bouma G.J. (2014). Regulation of ACVR1 and ID2 by Cell-Secreted Exosomes during Follicle Maturation in the Mare. Reprod. Biol. Endocrinol..

[39] Hung W.-T., Hong X., Christenson L.K., McGinnis L.K. (2015). Extracellular Vesicles from Bovine Follicular Fluid Support Cumulus Expansion. Biol. Reprod..

[40] Hung W.-T., Navakanitworakul R., Khan T., Zhang P., Davis J.S., McGinnis L.K., Christenson L.K. (2017). Stage-Specific Follicular Extracellular Vesicle Uptake and Regulation of Bovine Granulosa Cell Proliferation. Biol. Reprod..

[41] Gabryś J., Kij-Mitka B., Sawicki S., Kochan J., Nowak A., Łojko J., Karnas E., Bugno-Poniewierska M. (2022). Extracellular Vesicles from Follicular Fluid May Improve the Nuclear Maturation Rate of In Vitro Matured Mare Oocytes. Theriogenology.

[42] Lange-Consiglio A., Perrini C., Albini G., Modina S., Lodde V., Orsini E., Esposti P., Cremonesi F. (2017). Oviductal Microvesicles and Their Effect on In Vitro Maturation of Canine Oocytes. Reproduction.

[43] Matsuno Y., Onuma A., Fujioka Y.A., Yasuhara K., Fujii W., Naito K., Sugiura K. (2017). Effects of Exosome-like Vesicles on Cumulus Expansion in Pigs In Vitro. J. Reprod. Dev..

[44] Mateo-Otero Y., Yeste M., Roca J., Llavanera M., Bucci D., Galeati G., Spinaci M., Barranco I. (2022). Seminal Extracellular Vesicles Subsets Modulate Gene Expression in Cumulus Cells of Porcine In Vitro Matured Oocytes. Sci. Rep..

[45] Diaz F.J., O’Brien M.J., Wigglesworth K., Eppig J.J. (2006). The Preantral Granulosa Cell to Cumulus Cell Transition in the Mouse Ovary: Development of Competence to Undergo Expansion. Dev. Biol..

[46] Liu Z., Fan H.-Y., Wang Y., Richards J.S. (2010). Targeted Disruption of Mapk14 (P38MAPKα) in Granulosa Cells and Cumulus Cells Causes Cell-Specific Changes in Gene Expression Profiles That Rescue COC Expansion and Maintain Fertility. Mol. Endocrinol..

[47] Fan H.-Y., Sun Q.-Y. (2004). Involvement of Mitogen-Activated Protein Kinase Cascade During Oocyte Maturation and Fertilization in Mammals1. Biol. Reprod..

[48] Su Y.-Q., Denegre J.M., Wigglesworth K., Pendola F.L., O’Brien M.J., Eppig J.J. (2003). Oocyte-Dependent Activation of Mitogen-Activated Protein Kinase (ERK1/2) in Cumulus Cells Is Required for the Maturation of the Mouse Oocyte–Cumulus Cell Complex. Dev. Biol..

[49] Cibelli J.B., Iager A.E., Otu H.H. (2009). Genes Differentially Expressed by Cumulus Cells and Assays Using Same to Dentify Pregnancy Competent Oocytes. Faculty Publications from the Department of Electrical and Computer Engineering. http://digitalcommons.unl.edu/electricalengineeringfacpub/444.

[50] Salhab M., Tosca L., Cabau C., Papillier P., Perreau C., Dupont J., Mermillod P., Uzbekova S. (2011). Kinetics of Gene Expression and Signaling in Bovine Cumulus Cells throughout IVM in Different Mediums in Relation to Oocyte Developmental Competence, Cumulus Apoptosis and Progesterone Secretion. Theriogenology.

[51] Hu C.-L., Cowan R.G., Harman R.M., Quirk S.M. (2004). Cell Cycle Progression and Activation of Akt Kinase Are Required for Insulin-Like Growth Factor I-Mediated Suppression of Apoptosis in Granulosa Cells. Mol. Endocrinol..

[52] Yuan Y.Q., Van Soom A., Leroy J.L.M.R., Dewulf J., Van Zeveren A., de Kruif A., Peelman L.J. (2005). Apoptosis in Cumulus Cells, but Not in Oocytes, May Influence Bovine Embryonic Developmental Competence. Theriogenology.

[53] Lee K.S., Joo B.S., Na Y.J., Yoon M.S., Choi O.H., Kim W.W. (2001). Cumulus Cells Apoptosis as an Indicator to Predict the Quality of Oocytes and the Outcome of IVF-ET. J. Assist. Reprod. Genet..

[54] Abate C., Marshak D.R., Curran T. (1991). Fos Is Phosphorylated by P34cdc2, CAMP-Dependent Protein Kinase and Protein Kinase C at Multiple Sites Clustered within Regulatory Regions. Oncogene.

[55] Tanos T., Marinissen M.J., Leskow F.C., Hochbaum D., Martinetto H., Gutkind J.S., Coso O.A. (2005). Phosphorylation of C-Fos by Members of the P38 MAPK Family. J. Biol. Chem..

[56] Angel P., Karin M. (1991). The Role of Jun, Fos and the AP-1 Complex in Cell-Proliferation and Transformation. Biochim. Biophys. Acta-Rev. Cancer.

[57] Ohki M., Ohki Y., Ishihara M., Nishida C., Tashiro Y., Akiyama H., Komiyama H., Lund L.R., Nitta A., Yamada K. (2010). Tissue Type Plasminogen Activator Regulates Myeloid-Cell Dependent Neoangiogenesis during Tissue Regeneration. Blood.

[58] Li H., Zhou J., Wei X., Chen R., Geng J., Zheng R., Chai J., Li F., Jiang S. (2016). MiR-144 and Targets, c-Fos and Cyclooxygenase-2 (COX2), Modulate Synthesis of PGE2 in the Amnion during Pregnancy and Labor. Sci. Rep..

[59] Stocco C., Telleria C., Gibori G. (2007). The Molecular Control of Corpus Luteum Formation, Function, and Regression. Endocr. Rev..

[60] Perugini M., Kok C.H., Brown A.L., Wilkinson C.R., Salerno D.G., Young S.M., Diakiw S.M., Lewis I.D., Gonda T.J., D’Andrea R.J. (2009). Repression of Gadd45α by Activated FLT3 and GM-CSF Receptor Mutants Contributes to Growth, Survival and Blocked Differentiation. Leukemia.

[61] Chang B., Liu G., Yang G., Mercado-Uribe I., Huang M., Liu J. (2009). REDD1 Is Required for RAS-Mediated Transformation of Human Ovarian Epithelial Cells. Cell Cycle.

[62] Goudet G., Belin F., Bezard J., Gerard N. (1998). Maturation-Promoting Factor (MPF) and Mitogen Activated Protein Kinase (MAPK) Expression in Relation to Oocyte Competence for in-Vitro Maturation in the Mare. Mol. Hum. Reprod..

[63] Hinrichs K. (2010). The Equine Oocyte: Factors Affecting Meiotic and Developmental Competence. Mol. Reprod. Dev..

[64] Andrews S.C., Wood M.D., Tunster S.J., Barton S.C., Surani M.A., John R.M. (2007). Cdkn1c (P57 Kip2) Is the Major Regulator of Embryonic Growth within Its Imprinted Domain on Mouse Distal Chromosome 7. BMC Dev. Biol..

[65] Spate L.D., Brown A.N., Redel B.K., Whitworth K.M., Murphy C.N., Prather R.S. (2014). Dickkopf-Related Protein 1 Inhibits the WNT Signaling Pathway and Improves Pig Oocyte Maturation. PLoS ONE.

[66] Harwood B.N., Cross S.K., Radford E.E., Haac B.E., De Vries W.N. (2008). Members of the WNT Signaling Pathways Are Widely Expressed in Mouse Ovaries, Oocytes, and Cleavage Stage Embryos. Dev. Dyn..

[67] Schlesinger A., Shelton C., Maloof J., Meneghini M., Bowerman B. (1999). Wnt Pathway Components Orient a Mitotic Spindle in the Early Caenorhabditis Elegans Embryo without Requiring Gene Transcription in the Responding Cell. Genes. Dev..

[68] Posor Y., Eichhorn-Grünig M., Haucke V. (2015). Phosphoinositides in Endocytosis. Biochim. Et Biophys. Acta (BBA)-Mol. Cell Biol. Lipids.

[69] Nowak A., Kochan J., Witarski W., Okólski A. (2021). In Vitro Maturation of Equine Oocytes Followed by Two Vitrification Protocols and Subjected to Either Intracytoplasmic Sperm Injection (ICSI) or Parthenogenic Activation. Theriogenology.

[70] Takasugi M. (1971). An Improved Fluoroghromatic Cytotoxic Test. Transplantation.

[71] Chomczynski P., Sacchi N. (2006). The Single-Step Method of RNA Isolation by Acid Guanidinium Thiocyanate–Phenol–Chloroform Extraction: Twenty-Something Years On. Nat. Protoc..

[72] Maisarah Y., Hashida H.N., Yusmin M.-Y. (2020). The Challenge of Getting a High Quality of RNA from Oocyte for Gene Expression Study. Vet. Res. Forum.

[73] Dodt M., Roehr J., Ahmed R., Dieterich C. (2012). FLEXBAR—Flexible Barcode and Adapter Processing for Next-Generation Sequencing Platforms. Biology.

[74] Dobin A., Davis C.A., Schlesinger F., Drenkow J., Zaleski C., Jha S., Batut P., Chaisson M., Gingeras T.R. (2013). STAR: Ultrafast Universal RNA-Seq Aligner. Bioinformatics.

[75] Aken B.L., Ayling S., Barrell D., Clarke L., Curwen V., Fairley S., Fernandez Banet J., Billis K., García Girón C., Hourlier T. (2016). The Ensembl Gene Annotation System. Database.

[76] Anders S., Huber W. (2010). Differential Expression Analysis for Sequence Count Data. Nat. Preced..

[77] Thomas P.D., Ebert D., Muruganujan A., Mushayahama T., Albou L., Mi H. (2022). PANTHER: Making Genome-scale Phylogenetics Accessible to All. Protein Sci..

[78] Ge S.X., Jung D., Yao R. (2020). ShinyGO: A Graphical Gene-Set Enrichment Tool for Animals and Plants. Bioinformatics.

[79] Bu D., Luo H., Huo P., Wang Z., Zhang S., He Z., Wu Y., Zhao L., Liu J., Guo J. (2021). KOBAS-i: Intelligent Prioritization and Exploratory Visualization of Biological Functions for Gene Enrichment Analysis. Nucleic Acids Res..

[80] Vandesompele J., De Preter K., Pattyn F., Poppe B., Van Roy N., De Paepe A., Speleman F. (2002). Accurate Normalization of Real-Time Quantitative RT-PCR Data by Geometric Averaging of Multiple Internal Control Genes. Genome Biol..

[81] Pfaffl M.W. (2001). A New Mathematical Model for Relative Quantification in Real-Time RT-PCR. Nucleic Acids Res..

