# Correction: Gabryś et al. Follicular Fluid-Derived Extracellular Vesicles Influence on In Vitro Maturation of Equine Oocyte: Impact on Cumulus Cell Viability, Expansion and Transcriptome. *Int. J. Mol. Sci.* 2024, *25*, 3262

**DOI:** 10.3390/ijms25136812

**Published:** 2024-06-21

**Authors:** Julia Gabryś, Artur Gurgul, Tomasz Szmatoła, Barbara Kij-Mitka, Aneta Andronowska, Elżbieta Karnas, Mirosław Kucharski, Joanna Wojciechowska-Puchałka, Joanna Kochan, Monika Bugno-Poniewierska

**Affiliations:** 1 Department of Animal Reproduction, Anatomy and Genomics, Faculty of Animal Science, University of Agriculture in Krakow, Mickiewicza 24/28, 30-059 Krakow, Poland; julia.gabrys@urk.edu.pl (J.G.); barbara.kij-mitka@urk.edu.pl (B.K.-M.); joanna.wojciechowska-puchalka@urk.edu.pl (J.W.-P.); joanna.kochan@urk.edu.pl (J.K.); monika.bugno-poniewierska@urk.edu.pl (M.B.-P.); 2 Center for Experimental and Innovative Medicine, University of Agriculture in Krakow, Rędzina 1c, 30-248 Krakow, Poland; tomasz.szmatola@urk.edu.pl; 3 Institute of Animal Reproduction and Food Research, Polish Academy of Sciences, Tuwima 10, 10-748 Olsztyn, Poland; a.andronowska@pan.olsztyn.pl; 4 Department of Cell Biology, Faculty of Biochemistry, Biophysics and Biotechnology, Jagiellonian University, Gronostajowa 7, 30-387 Krakow, Poland; e.karnas@uj.edu.pl; 5 Department of Animal Physiology and Endocrinology, University of Agriculture in Krakow, Mickiewicza 24/28, 30-059 Krakow, Poland; miroslaw.kucharski@urk.edu.pl

In the original publication [1], there was a mistake in the figure, the legend, and the text related to the results and methodology associated with ffEV characterization using nanoparticle tracking analysis (NTA). The mistake concerns Figure 1B, encompassing a graphical depiction of NTA results and a table detailing the average size of extracted extracellular vesicles, the D90 parameter, and particle concentration. The error also relates to the estimated efficiency of particle extraction from the follicular fluid, as well as details regarding the equipment utilized for NTA. 

The correct Figure 1, its legend, and text appear below. 

**Figure 1:**
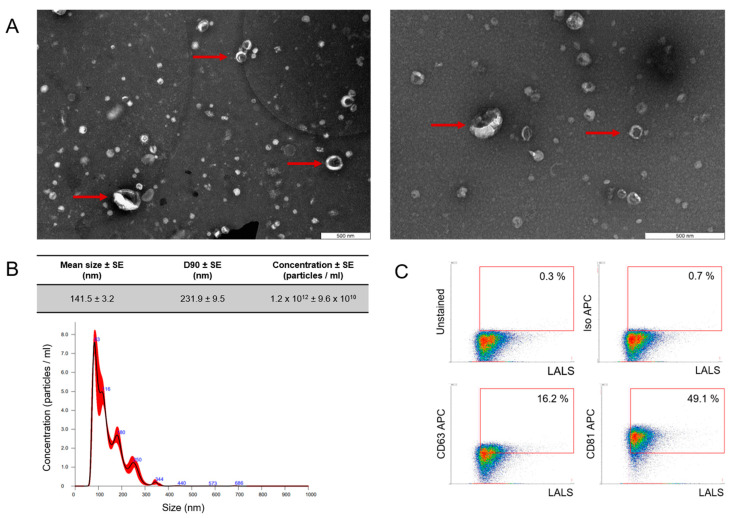
Characterization of follicular fluid-derived EVs. (**A**) EVs purified from follicular fluid were analyzed by transmission electron microscopy, where the red arrow points toward the presence of EVs. (**B**) The size profile was measured by NanoSight NTA, which revealed one population of ffEVs with a mean diameter of 141.5 nm. D90—value indicates the percentage of particles (90%) less than or equal to the appropriate mean particle size. The red bars represent the standard error of the mean, while the blue numbers denote the means of clusters. (**C**) Density plot of EVs purified from follicular fluid showed a positive signal for EV-specific markers CD63 and CD81. To validate the specificity of the data obtained, APC isotype and unstained control were incorporated into the gating strategy.

A correction has been made to **2. Results**, *2.1. ffEV Characterization*, Paragraph Number 1:

FF-derived EVs were characterized using nanoparticle tracking analysis (NTA), transmission electron microscopy (TEM), and flow cytometry (FC). Physical analysis using TEM showed the presence of particles with the cup shape that is characteristic of EVs, as indicated by the arrows (Figure 1A). NTA showed that most of the molecules obtained were within the limits of “small EVs” [25], with a mean particle size of 141.5 nm, and a D90 value of 231.9 nm, signifying that 90% of the particles were equal to or smaller than the appropriate mean particle size [25] (Figure 1B). The mean EV concentration was 1.2 × 10^12^ +/− 9.6 × 10^10^ and the efficacy in acquiring EVs from follicular fluid reached approximately 6 × 10^9^ particles/mL. Phenotypical analysis with high-resolution FC confirmed that the isolated particles expressed EV-specific surface markers (Figure 1C) at the level of 16.2% and 49.1% for CD63 and CD81, respectively.

A correction has been made to **4. Materials and Methods**, *4.6. Nanoparticle Tracking Analysis (NTA)*, Paragraph Number 1:

Particle concentration and size distribution in ffEV samples were assessed by NanoSight NS300 3.4 Build 3.4.003 analytical software (Malvern Instruments, Malvern, UK) employing nanoparticle tracking analysis (NTA). Follicular EV preparations were diluted before analysis in 0.22 µm filtered DPBS without Ca^2+^ or Mg^2+^ (Lonza, Basel, Switzerland). Each sample underwent three 1 min recordings with an sCMOS camera level set at 13. The recordings were conducted at a consistent temperature of 20 °C, producing three histograms for each sample, which were then averaged. The D90 parameter, where 90% of the EV population had a diameter equal to or less than the reported mean value, was also determined.

The authors state that the scientific conclusions are unaffected. This correction was approved by the Academic Editor. The original publication has also been updated.
