# Supplementary material for: Follicular Fluid-Derived Extracellular Vesicles Influence on In Vitro Maturation of Equine Oocyte: Impact on Cumulus Cell Viability, Expansion and Transcriptome
Source: Int J Mol Sci. 2024 Mar 13;25(6):3262. doi: 10.3390/ijms25063262 (PMC10970002; doi:10.3390/ijms25063262)
Supplement: Supplementary file 1 [file ijms-25-03262-s001.zip › Table S1.pdf]

Table S1. Average change in COC dimensions.

Group	Culture no.	Mean H0 [um]	Mean H38 [um]	Expansion [%]	Mean Group H0 [um]	Mean Group H38 [um]	Mean Group Expansion [%]
CP + ffEVs	1	1133.9	1432.51	26.33	861.94[a]	1499.55[b]	78.83
	2	694.45	1704.15	145.4			
	3	744.92	1340.26	79.92			
	4	843.71	1594.74	89.02			
	5	845.26	1269.255	50.16			
	6	909.385	1656.37	82.14			
CP control	1	857.93	714.22	−16.75	801.77	917.62	15.81
	2	705.2	990.66	40.48			
	3	844.12	846.98	0.34			
	4	828.52	1092.57	31.87			
	5	823.81	758.41	−7.94			
	6	751.05	1102.86	46.84			
EX + ffEVs	1	627.98	779.65	24.15	741.34[a]	1044.87[b]	40.37
	2	733.02	812.58	10.85			
	3	764.92	970.695	26.9			
	4	777.43	1022.92	31.58			
	5	726.92	1349.66	85.67			
	6	817.79	1333.69	63.08			
EX control	1	579.23	484.01	−16.44	719.67	819.1	11.56
	2	602.17	695.31	15.47			
	3	741.72	925.03	24.71			
	4	689.36	708.63	2.8			
	5	734.95	796.27	8.34			
	6	970.58	1305.35	34.49			

[a,b]—different letters in the index indicate statistically significant differences between columns
