# Supplementary material for: Follicular Fluid-Derived Extracellular Vesicles Influence on In Vitro Maturation of Equine Oocyte: Impact on Cumulus Cell Viability, Expansion and Transcriptome
Source: Int J Mol Sci. 2024 Mar 13;25(6):3262. doi: 10.3390/ijms25063262 (PMC10970002; doi:10.3390/ijms25063262)
Supplement: Supplementary file 1 [file ijms-25-03262-s001.zip › Table S4.pdf]

Table S4. Selected biological processes, molecular functions and cellular components enriched in DE genes (adjP < 0.1) in CC of Cp COCs after 12 h treatment with ffEVs. GO – Gene Ontology term; P – adjusted *p*-value.

GO	Description	Term ID	FDR	Engaged genes
Biological processes	cell cycle	GO:0007049	0.004	*GPNMB, PARD6B, NUSAP1, SEPTIN9, ASPM, JUN, CENPF, KIFC1, GADD45A*
	organelle localization	GO:0051640	0.004	*VMP1, KIF5C, NUSAP1, ASPM, CENPF, KIFC1*
	microtubule-based process	GO:0007017	0.009	*PARD6B, CEP57, KIF5C, NUSAP1, GAS8, ASPM, KIFC1*
	microtubule cytoskeleton organization	GO:0000226	0.010	*PARD6B, CEP57, NUSAP1, GAS8, ASPM, KIFC1*
	cell cycle process	GO:0022402	0.015	*GPNMB, PARD6B, NUSAP1, SEPTIN9, ASPM, CENPF, KIFC1*
	response to muscle stretch	GO:0035994	0.018	*FOS, JUN*
	chromosome segregation	GO:0007059	0.030	*CIAO2A, NUSAP1, CENPF, KIFC1*
	response to mechanical stimulus	GO:0009612	0.030	*FOS, JUN, GADD45A*
	negative regulation of phosphorylation	GO:0042326	0.030	*DDIT4, DUSP1, JUN, GADD45A*
	negative regulation of nucleobase-containing compound metabolic process	GO:0045934	0.030	*DDIT4, HMGB2, JUN, CENPF, MAF, FOXL2, GADD45A*
Molecular functions	microtubule binding	GO:0008017	6.21×10^{-5}	*CEP57, KIF5C, NUSAP1, GAS8, CENPF, KIFC1*
	tubulin binding	GO:0015631	1.70×10^{-4}	*CEP57, KIF5C, NUSAP1, GAS8, CENPF, KIFC1*
	r-smad binding	GO:0070412	0.015	*FOS, JUN*
	cytoskeletal protein binding	GO:0008092	0.015	*CEP57, KIF5C, NUSAP1, GAS8, CENPF, KIFC1*
	transcription factor binding	GO:0008134	0.041	*HMGB2, FOS, JUN, CENPF*
Cellular components	microtubule	GO:0005874	6.3×10^{-6}	*CEP57, KIF5C, NUSAP1, GAS8, SEPTIN9, ASPM, KIFC1*
	polymeric cytoskeletal fiber	GO:0099513	1.1×10^{-4}	*CEP57, KIF5C, NUSAP1, GAS8, SEPTIN9, ASPM, KIFC1*
	transcription factor ap-1 complex	GO:0035976	2.3×10^{-4}	*FOS, JUN*
	supramolecular complex	GO:0099080	2.3×10^{-4}	*CEP57, KIF5C, NUSAP1, GAS8, SEPTIN9, ASPM, CENPF, KIFC1*
	supramolecular polymer	GO:0099081	2.3×10^{-4}	*CEP57, KIF5C, NUSAP1, GAS8, SEPTIN9, ASPM, KIFC1*
	supramolecular fiber	GO:0099512	2.3×10^{-4}	*CEP57, KIF5C, NUSAP1, GAS8, SEPTIN9, ASPM, KIFC1*
	microtubule cytoskeleton	GO:0015630	6.2×10^{-4}	*CEP57, KIF5C, NUSAP1, GAS8, SEPTIN9, ASPM, CENPF, KIFC1*
	spindle	GO:0005819	0.007	*NUSAP1, ASPM, CENPF, KIFC1*
	mitotic spindle	GO:0072686	0.008	*NUSAP1, ASPM, KIFC1*
	plasma membrane bounded cell projection cytoplasm	GO:0032838	0.010	*KIF5C, GAS8, SEPTIN9*
