# Supplementary material for: Follicular Fluid-Derived Extracellular Vesicles Influence on In Vitro Maturation of Equine Oocyte: Impact on Cumulus Cell Viability, Expansion and Transcriptome
Source: Int J Mol Sci. 2024 Mar 13;25(6):3262. doi: 10.3390/ijms25063262 (PMC10970002; doi:10.3390/ijms25063262)
Supplement: Supplementary file 1 [file ijms-25-03262-s001.zip › Table S6.pdf]

Table S6. Selected biological processes, molecular functions, and cellular components enriched in DE genes (adjP < 0.1) in CC of Ex COCs after 12 h treatment with ffEVs. GO – Gene Ontology term; P – adjusted *p*-value.

GO	Description	Term ID	FDR	Engaged Genes
Biological processes	female meiotic nuclear division	GO:0007143	0.024	*TOP2A, TTK*
	regulation of mitotic cell cycle	GO:0007346	0.024	*GPNMB, TTK, CENPF, CDKN1C*
	meiotic chromosome separation	GO:0051307	0.024	*TOP2A, TTK*
	negative regulation of mitotic cell cycle	GO:0045930	0.024	*GPNMB, TTK, CDKN1C*
	regulation of mitotic cell cycle phase transition	GO:1901990	0.034	*GPNMB, TTK, CENPF*
	phosphorylation	GO:0016310	0.034	*GPNMB, FZD5, DAB2, ITPKA, TTK, CDKN1C*
	regulation of cell cycle phase transition	GO:1901987	0.042	*GPNMB, TTK, CENPF*
	cell cycle	GO:0007049	0.042	*GPNMB, TOP2A, TTK, CENPF, CDKN1C*
	chromosome segregation	GO:0007059	0.042	*TOP2A, TTK, CENPF*
	mitotic cell cycle	GO:0000278	0.042	*GPNMB, TTK, CENPF, CDKN1C*
Molecular functions	protein c-terminus binding	GO:0008022	0.005	*TOP2A, DAB2, CENPF*
Cellular components	chromosome, centromeric region	GO:0000775	0.006	*TOP2A, TTK, CENPF*
	condensed chromosome	GO:0000793	0.006	*TOP2A, TTK, CENPF*
	chromosomal region	GO:0098687	0.007	*TOP2A, TTK, CENPF*
	kinetochore	GO:0000776	0.025	*TTK, CENPF*
	condensed chromosome, centromeric region	GO:0000779	0.025	*TTK, CENPF*
	outer kinetochore	GO:0000940	0.048	*CENPF*
	ciliary transition fiber	GO:0097539	0.048	*CENPF*
