# Supplementary material for: Follicular Fluid-Derived Extracellular Vesicles Influence on In Vitro Maturation of Equine Oocyte: Impact on Cumulus Cell Viability, Expansion and Transcriptome
Source: Int J Mol Sci. 2024 Mar 13;25(6):3262. doi: 10.3390/ijms25063262 (PMC10970002; doi:10.3390/ijms25063262)
Supplement: Supplementary file 1 [file ijms-25-03262-s001.zip › Table S8.pdf]

Table S8. Correlation coefficients between gene expression levels determined with qPCR and RNA-Seq methods.

**Gene**	**Pearson**		**Spearman**	
	r	*p*	rho	*p*
CENPF	−0.260	0.415	−0.014	0.974
DDIT4	0.650	0.114	0.393	0.396
HAS2	0.611	0.061	0.370	0.296
