# Supplementary material for: Follicular Fluid-Derived Extracellular Vesicles Influence on In Vitro Maturation of Equine Oocyte: Impact on Cumulus Cell Viability, Expansion and Transcriptome
Source: Int J Mol Sci. 2024 Mar 13;25(6):3262. doi: 10.3390/ijms25063262 (PMC10970002; doi:10.3390/ijms25063262)
Supplement: Supplementary file 1 [file ijms-25-03262-s001.zip › Table S9.pdf]

Table S9. Target and housekeeping genes primers.

Gene	Relevance	Strand	Sequence (5'-3')	Product size [bp]	Reference (NCBI)
HAS2	hyaluronan synthase 2	F	tcctggtgagacggatgagt	193	NM_001081801.2
		R	ggcagggtcaagcatggtat		
DDIT4	DNA damage-inducible transcript 4 protein	F	cttcgtcgtcctcctcttcg	196	XM_001502859.5
		R	ccatccaggtatgccgagtc		
CENPF	centromere protein F	F	ggcctcgttcaagaagtgga	97	XM_023640730.1
		R	gctgctcttggtcttggagt		
GAPDH	glyceraldehyde-3-phosphate dehydrogenase	F	ggactcatgaccacagtgca	109	NM_001163856.1
		R	cagggatgatgttctgggca		
YWHAZ	tyrosine 3-monooxygenase	F	ggtcatcttggagggtcgtc	186	XM_001492988.6
		R	agaagactttgctctccgct		
